# Supplementary material for: Exploring the relationship between frequent internet use and health and social care resource use in a community-based cohort of older adults: an observational study in primary care
Source: BMJ Open. 2017 Jul 21;7(7):e015839. doi: 10.1136/bmjopen-2017-015839 (PMC5642753; doi:10.1136/bmjopen-2017-015839)
Supplement: Supplementary Appendix 1 [file bmjopen-2017-015839supp001.pdf]

Appendix

A section of the Service use diary completed by participants at baseline, 3 and 6 months. The binary Y/N question for section D was a new variable generated by assigning a "Yes" for any participant who responded "Yes" to any sub-questions in that section.

Diary Two

We would like to know about any contact you have had with services, organisations or others about your health and well-being.

Please fill in the number of contacts you have had **during the last three months** for each question listed below. If you haven't had any contact with them, please fill in a 0 (zero).

**A.** Have you needed to go to **hospital in the last 3 months?**<br>Yes ☐ Complete 1-4 below<br>No ☐ Go to Section B	
1. How many nights have you spent in hospital as an inpatient?	
2. How many days have you been admitted to a day ward in hospital without staying overnight?	
3. How many times have you had an appointment with a doctor in the hospital or have you attended a clinic appointment?	
4. How many times have you been treated at Casualty or Accident and Emergency?	
**B.** Have you seen/spoken your **GP or community nurse in the last 3 months**?<br>Yes ☐ Complete 1-7 below<br>No ☐ Go to Section C	
1. How many times have you visited your GP at their surgery or health centre?	
2. How many times has a GP visited you at home?	
3. How many times have you spoken to a GP on the phone?	
4. How many times has a nurse visited you at home?	
5. How many times have you visited a nurse at the surgery or health centre?	
6. How many times have you spoken to a nurse on the phone?	
7. How many times have you called NHS Direct?	

ID:

**C.** Have you contacted/been to **any other health care services in the last 3 months?**<br>Yes ☐ Complete 1-10 below<br>No ☐ Go to Section D	
1. How many times have you seen a physiotherapist? (please indicate if NHS or private) …………………………………………..	
2. How many times have you seen an osteopath or chiropractor (please indicate if NHS or private)?………………………………	
3. How many times have you seen a dentist (please indicate if NHS or private)?……………………………………………………	
4. How many times have you seen someone about your sight (eg optician / optometrist?)	
5. How many times have you seen someone about your hearing (eg an audiologist/ or an ear/hearing clinic?)	
6. How many times have you been to a counselling service?	
7. How many times have you been to a smoking cessation service?	
8. How many times have you been to a chiropodist or podiatrist (foot) clinic?	
9. How many times have you contacted any emergency services (Police, Ambulance, Fire)? Please give details …………………………………………………………………………	
10. How many times have you used *another* health service contact? e.g. falls prevention service. Please give details ………………………………………………………………………………	

D. Personal care
*Please think about an average or typical week during the **last 3 months***

Have you had any **paid** help (including both help arranged or paid for by social services and help arranged/paid for privately) with:

1. Washing, dressing or having a bath/shower

Yes ☐ No ☐ If yes, how many times *per week*? …………...

2. Cooking/preparing meals or shopping

Yes ☐ No ☐ If yes, how many times *per week*? ……………

3. Meals on wheels or other meal delivery service

Yes ☐ No ☐ If yes, how many times *per week*? …………...

4. Other (eg laundry, sitting service): please give details and number of times *per*

ID:

Have you had any **unpaid** help e.g. from family or friends, with:

5. Washing, dressing or having a bath/shower

Yes ☐ No ☐ If yes, how many times *per week*? ……………

6. Cooking/preparing meals or shopping

Yes ☐ No ☐ If yes, how many times *per week*? …………...

7. Other (eg laundry, sitting service): please give details and number of times *per week*?

……………………………………………………………………………………………...
