# Supplementary material for: Exploring the relationship between frequent internet use and health and social care resource use in a community-based cohort of older adults: an observational study in primary care
Source: BMJ Open. 2017 Jul 21;7(7):e015839. doi: 10.1136/bmjopen-2017-015839 (PMC5642753; doi:10.1136/bmjopen-2017-015839)
Supplement: Author's manuscript [file bmjopen-2017-015839.draft_revisions.pdf]

BMJ Open

Exploring the relationship between frequent internet use and health and social care resource use by older adults

Journal:	*BMJ Open*
Manuscript ID	bmjopen-2017-015839
Article Type:	Research
Date Submitted by the Author:	04-Jan-2017

Complete List of Authors:	Clarke, Caroline; University College London, Research Department of Primary Care and Population Health<br>Round, Jeff; University of Bristol School of Social and Community Medicine<br>Morris, Stephen; University College London, Department of Applied Health Research<br>Kharicha, Kalpa; University College London, Primary Care and Population Health<br>Ford, John; University of East Anglia, Public Health<br>Manthorpe, Jill; King's College London, Social Care Workforce Research Unit<br>Iliffe, Steve; University College London, Primary Care and Population Sciences<br>Goodman, Claire; University of Hertfordshire, Centre for Research in Primary and Community Care<br>Walters, Kate; University College London, Primary Care and Population Health

**Primary Subject Heading**:	General practice / Family practice
Secondary Subject Heading:	Health services research, Geriatric medicine
Keywords:	Older adults, Health service resource use, Internet use, Panel data, Logistic regression, PRIMARY CARE

SCHOLARONE™ Manuscripts

Exploring the relationship between frequent internet use and health and social care resource use by older adults

Caroline S. Clarke[1], Jeff Round[2], Stephen Morris[3], Kalpa Kharicha[1], John Ford[4], Jill Manthorpe[5], Steve Iliffe[1]*, Claire Goodman[6], Kate Walters[1]

Affiliations:

[1] UCL Research Dept. of Primary Care & Population Health, UCL Medical School, Upper 3rd Floor, Royal Free Campus, Rowland Hill St, London, NW3 2PF, UK

[2] School of Social and Community Medicine, University of Bristol, Canynge Hall, 39 Whatley Road, Bristol, BS8 2PS, UK

[3] Department of Applied Health Research, University College London (UCL), 1-19 Torrington Place, London, WC1E 7HB, UK

[4] Department of Public Health and Primary Care, Norwich Medical School, University of East Anglia, Chancellors Drive, Norwich, NR4 7TJ, UK

[5] Social Care Workforce Research Unit, King's College London, Strand, London, WC2 4LF, UK

[6] Centre for Research in Primary and Community Care, University of Hertfordshire, AL10 9AB, UK

* Emeritus

Corresponding author:

Email: caroline.clarke@ucl.ac.uk

Tel.: +44 (0)20 7794 0500 ext. 36770

Word count: 2,570

For peer review only

Abstract

Objectives

Given many countries' ageing populations, policymakers must consider how to mitigate or reduce health problems associated with old age, within budgetary constraints. Evidence of use of digital technology in delaying the onset of illness and reducing healthcare service use is mixed, and no clear consensus has yet been formed. Our aim was to investigate the relationship between frequent internet use and patterns of health or social care resource use in primary care attendees who took part in a study to improve the health of older adults.

Methods

Participants, aged over 65 and living in semi-rural or urban areas in the south of England, were followed up at 3 and 6 months after completing a comprehensive questionnaire with personalised feedback on their health and well-being. We performed logistic regression analyses to investigate relationships between frequent internet use and patterns of service use, controlling for confounding factors, and clustering by GP practice. Four categories of service use data were gathered: use of primary NHS care; secondary NHS care; other community health and social care services; and assistance with washing, shopping and meals.

Results

Our results show, in this relatively healthy population, a positive relationship (odds ratio 1.72; 95% CI 1.33 to 2.23) between frequent internet use and use of any other community-based health services (physiotherapist, osteopath/chiropractor, dentist, optician/optometrist, counselling service, smoking cessation service, chiropodist/ podiatrist, emergency services, other non-specific health services), and no relationship with the other types of care. No causal relationship can be postulated due to the study's design.

Conclusions

No observed relationship between frequent internet use and primary or secondary care use was found, suggesting that older adults without internet access are not disadvantaged regarding health care utilisation. Further research should explore how older people use the internet to access healthcare, and impact on their health.

Key words: Older adults, health service resource use, internet use, panel data, logistic regression, primary care.

For peer review only

Article summary

Strengths and limitations of this study

Strengths:

- Timely study providing an update on older adults' use of the internet at home.
- Lack of bias: the question on internet use was one of many, so participants will not have attached much weight to it and are therefore likely to have been more honest in answering it.

Limitations:

- We cannot speculate on how much of the internet use was specifically for looking up information on health or accessing health-related services, as opposed to general correspondence, or looking up information on any other non-health services, for example.
- Causality cannot be inferred.
- Small study size (n=454)

Funding: The WISH study was funded by the Medical Research Council (MRC) LLHW G1001822/1. The MRC had no role in the design, collection, analysis, or interpretation of data; in the writing of this manuscript; or in the decision to submit the manuscript for publication. London-East Research Ethics Committee approved the WISH study (reference 11/LO/1814). The corresponding author for this analysis was not involved in the WISH study and received no specific grant from any funding agency in the public, commercial or not-for-profit sectors.

Conflict of Interest: All authors have completed the ICMJE uniform disclosure form at www.icmje.org/coi_disclosure.pdf and declare: no support from any organisation for the submitted work; no financial relationships with any organisations that might have an interest in the submitted work in the previous three years; no other relationships or activities that could appear to have influenced the submitted work.

Data sharing statement: no additional data available.

For peer review only

Introduction

Life expectancy is rising worldwide, and much research seeks to find ways of improving older people's health and well-being (1). Work has been undertaken by various groups regarding complex interventions designed to alter behaviour to improve health and well-being, enabling older adults to maintain their independence and good health for longer, however there is no clear consensus on the best approaches (2) (3) (4) (5). It has been argued that the use of technology by older people could help in maintaining health and well-being and/or assist in managing or reducing health-related resource use (6) (7); similarly, other work has suggested that older adults might be disadvantaged if they do not use information and communications technology regularly (8).

There is significant use of the internet by older people, particularly by those in their 60s and 70s, but it is not universal and decreases with age (9). Research on how older people's use of the internet influences the way they seek help/use healthcare and other resources is still in its infancy (10) (11) (12) (13). There seem to be differences between how younger and older people use the internet; for example, older adults who use the internet seem to use it primarily for email, whereas a large proportion of younger people use social media sites, both for information and for socialising (14). As the majority of older people have multi-morbidities with increasingly complex health and other needs (15), this might influence their use of the internet in relation to their health, as well as there being differences in digital and health literacy in comparison to younger sections of the population.

The WISH (Well-being Interventions for Social and Health needs) study (16) (17) assessed the feasibility of implementing a risk appraisal system, the Multi-dimensional Risk Appraisal in Older people (MRA-O), for older adults living at home (16) (17). It recruited older adults from English primary care settings in the London Borough of Ealing (urban) and Hertfordshire (semi-rural) (16) (17). The MRA-O is an extension of the Health Risk Appraisal in Older people (HRA-O) system (18) (19) (20), including domains identified as having an impact on health and well-being in later life during the Smarter Working in Social and Health Care (SWISH) project (21) (22).

The MRA-O was administered as a postal questionnaire covering a broad range of health, lifestyle, social and environmental domains, including questions on internet use. Participants' responses were fed into a software system that generated personalised feedback and advice aimed at improving health and well-being and this was communicated to the participants and their GPs. Those with complex unmet needs were offered further consultations with their GP or practice nurse. Participants were followed up by the central

For peer review only

research team at 3 and 6 months to assess the impact of the MRA-O on their health and well-being, and to monitor resource use (16) (17). The resource use data included data on a wide range of services, both public and privately funded, and data on use of the internet.

The aim of the current study was to examine the relationship between frequent internet use and different types of health and social care resource use, and whether differences in internet use raise concerns about equity of access and use of care services.

Methods

Data collection

The WISH study collected a broad range of data from 454 participants aged 65 years and over from five general practices in two diverse areas, including physical and mental well-being, functional ability, lifestyle and diet, personal characteristics, loneliness and social networks, use of health care and social resources, and internet and mobile phone use (16) (17).

Measurements

Resource use

The WISH study measured resource use across a range of services, including primary and secondary healthcare, informal and other community health care, and support from informal carers or social services. These were captured in this analysis as four individual binary resource use variables, where "yes" meant that one or more of the difference types of contact listed below had occurred within the last three months:

A. Secondary care: hospital attendance (A&E, inpatient, outpatient)
B. Primary care: GP/community nurse consultation (by phone, face-to-face, a home visit, or a call to NHS Direct)
C. Other health care services (either NHS or private): physiotherapist, osteopath/chiropractor, dentist, optician/optometrist, hearing clinic/audiologist, counsellor, smoking cessation service, chiropodist/podiatrist, emergency services (police, ambulance, fire)
D. Wash/meals: any paid or unpaid help (e.g. from family member) with washing, dressing, having a bath/shower, cooking/preparing meals, shopping, or meal delivery service. The overall binary variable here returns a "yes" if any paid or unpaid help was reported.

Participants who responded 'Yes' were also asked sub-questions in each case, regarding how many contacts they had had with different services, e.g. how many nights did you stay in hospital, how many times did you speak with the general practice nurse on the phone. The complete list of questions can be found in the

For peer review only

Appendix. The principal binary questions for the resource variables were used instead of counting the numbers of contacts due to missing data in the sub-questions.

Internet use

The internet use question offered four possible answers: often (most days); sometimes (1-3 days a week); occasionally (less than once a week); and never. For the purposes of this analysis, it was dichotomised as “often/sometimes” vs. “occasionally/never” as the numbers of responses across the four groups were too small to allow meaningful analysis as a four-category variable.

Covariates

We controlled for a range of patient characteristics in the analyses: GP practice location (Ealing or Hertfordshire), season of study entry (summer or autumn), sex, age (in bands: 65-74 years, 75-84 years, 85+ years), ethnicity (White British or other), loneliness status (scoring 0-1 or 2-6 on the de Jong Gierveld 6-item short scale (23) corresponds to “not lonely” or “lonely”, respectively), Short Form SF-12 (24) mental health component summary score and physical health component summary score, occurrence of a recent sudden illness in the 3 months before baseline, and age at which left full-time education (before or after 17 years of age), which was used as a proxy variable for socioeconomic status. The simplicity of the ethnic group division chosen was due to low participant numbers in any non-White-British group, particularly at the semi-rural practices.

Analysis

We undertook logistic regression for each of the four dichotomous dependent outcome variables on service use, with the GP surgery contributing random effects. The covariates for the final multivariate regression models were chosen using the common model-selection criteria, the Akaike information criterion (AIC) and the Bayesian information criterion (BIC). We report odds ratios and 95% confidence intervals to investigate the relationship between frequent internet use and different types of resource use, controlling for patient characteristics described above.

The data were set as panel data using the patient ID code as the panel variable, and the number of months’ follow-up was set as the time variable (0, 3 and 6 months), although we note that exclusion of the time variable when setting the data led to no difference in the regression results. Our analysis considered complete cases only.

For peer review only

Results

Sample characteristics

Participants' use of the internet was asked as a four-category question: often, i.e. most days (44%); sometimes, i.e. 1-3 days a week (11%); occasionally, i.e. less than once a week (8%); and never (37%), and this was dichotomised as frequently (55%) and infrequently (45%). Patient characteristics, split by internet use, are given in Table 1. There was a large amount of missing data in the sub-questions regarding numbers of each specific type of contact in each of the four resource use types, with between 3.4% and 48.1% of those who responded "Yes" to the principal question failing to then state any numbers of contacts. The amount of missing data in the four principal binary service use variables was much smaller: the total number of participants in the WISH study at baseline was 454, dropping to 405 (89% retention) at the 3-month timepoint and 348 (77% retention) at 6 months. Only 4% or fewer participants who responded at each timepoint were excluded from the complete case analyses (see Table 2), and this was due to missing covariate data.

Table 1. Characteristics of participants, split according to whether or not they used the internet frequently (complete cases only).

Covariates		Using internet infrequently, n (%)	Using internet frequently, n (%)	Overall* n (%)
Site (semi-rural; other option was urban)		126/198 (63.6%)	154/247 (62.4%)	283/454 (62.3%)
Season at start (autumn; other option was summer)		94/198 (47.5%)	119/247 (48.2%)	216/454 (47.6%)
Gender (female)		116/198 (58.6%)	120/247 (48.6%)	240/454 (52.9%)
Age bands	65-74 years	88/198 (44.4%)	182/247 (73.7%)	272/454 (59.9%)
	75-84 years	84/198 (42.4%)	60/247 (24.3%)	151/454 (33.3%)
	85+ years	26/198 (13.1%)	5/247 (2.0%)	31/454 (6.8%)
White British		162/192 (84.4%)	215/246 (87.4%)	385/447 (86.1%)
Lonely (6-item de Jong Gierveld score)		67/173 (38.7%)	72/231 (31.2%)	142/407 (34.9%)
SF-12 mental score (mean, SD, [n])		52.6, 8.2 [179]	53.7, 8.9 [233]	53.2, 8.6 [412]
SF-12 physical score (mean, SD, [n])		40.6, 12.7 [179]	46.5, 11.7 [233]	43.9, 12.5 [412]
Recent sudden illness		35/192 (18.2%)	38/242 (15.7%)	75/442 (17.0%)
Left FT education before 17 years of age		145/196 (74.0%)	122/246 (49.6%)	274/451 (60.8%)

For peer review only

Table 2. Complete case numbers of yes and no responses for the four binary resource use variables.

Resource use variable	Baseline	3 months	6 months
(A) Secondary care – Yes	166/444 (37%)	161/403 (40%)	118/334 (35%)
– No	278/444 (63%)	242/403 (60%)	216/334 (65%)
(B) Primary care – Yes	327/443 (74%)	301/399 (75%)	235/333 (71%)
– No	116/443 (26%)	98/399 (25%)	98/333 (29%)
(C) Other health care – Yes	238/443 (54%)	227/401 (57%)	169/335 (50%)
– No	205/443 (46%)	174/401 (43%)	166/335 (50%)
(D) Wash/Meals – Yes	62/446 (14%)	72/403 (18%)	52/336 (15%)
– No	384/446 (86%)	331/403 (82%)	284/336 (85%)

Univariable unadjusted analyses

Shown in Table 3 are the raw unadjusted relationships between each of the covariates and each binary service use variable. These results show the relationship between each resource use variable and each covariate, with no controlling for age, sex, etc. Models with controlling variables included were constructed using the AIC and BIC, and gave an improved fit to the data compared to the univariable models. The multi-variable models' results are shown in Table 4 and outlined here below.

Multi-variable adjusted analyses

(A) Hospital use

When controlling for age, sex, site, season at start, SF-12 mental and physical component scores, having had a recent sudden illness, ethnicity, age at which left full-time education, and loneliness, there was no observed association between hospital use and frequent internet use (OR 0.98, 95% CI 0.25 to 3.84) (see Table 4).

(B) Primary care

Use of primary care services, controlling for all the above variables, was also not associated with frequent internet use (OR 1.15, 95% CI 0.78 to 1.70) (see Table 4).

(C) Other healthcare

Frequent internet use was, however, positively associated with use of other health care services (e.g. optician, dentist, physiotherapist, etc.) (OR 1.72, 95% CI 1.33 to 2.23) (see Table 4).

(D) Washing/meals assistance

For peer review only

Of those participants who stated that they were using assistance of this nature, approximately a quarter were using paid services. Receipt of assistance (paid or unpaid) for washing, cooking and similar tasks was not associated with frequent internet use (OR 0.56, 95% CI 0.12 to 2.55) (see Table 4).

Table 3. Odds ratios (95% confidence interval) from univariable unadjusted analyses of each individual covariate and its relationship to each resource use variable.

Independent variables		(A) Hospital use	(B) Primary care use	(C) Other health care services	(D) Wash/Meals assistance
Frequent internet use		0.76 (0.47 to 1.25)	0.79 (0.52 to 1.20)	1.42 (0.97 to 2.09)	0.12 (0.05 to 0.29)
Age bands	65-74 years	reference case	reference case	reference case	reference case
	75-84 years	1.44 (0.85 to 2.44)	1.54 (0.98 to 2.42)	0.94 (0.62 to 1.42)	7.36 (2.98 to 18.19)
	85+ years	1.55 (0.59 to 4.08)	4.76 (1.70 to 13.36)	1.50 (0.68 to 3.34)	176.38 (33.87 to 918.41)
Gender (male)		1.13 (0.69 to 1.84)	0.95 (0.62 to 1.43)	0.57 (0.39 to 0.83)	0.60 (0.25 to 1.41)
Site (urban)		1.35 (0.82 to 2.23)	0.95 (0.62 to 1.46)	1.40 (0.94 to 2.08)	2.76 (1.15 to 6.63)
Season at start (autumn)		0.71 (0.44 to 1.16)	0.71 (0.47 to 1.07)	0.84 (0.57 to 1.24)	2.26 (0.95 to 5.37)
SF-12 mental score		0.97 (0.94 to 1.00)	0.96 (0.93 to 0.98)	0.97 (0.94 to 0.99)	0.94 (0.89 to 0.99)
SF-12 physical score		0.94 (0.92 to 0.96)	0.95 (0.93 to 0.97)	0.98 (0.96 to 0.99)	0.85 (0.82 to 0.89)
Recent sudden illness		5.07 (2.68 to 9.59)	3.40 (1.79 to 6.46)	3.46 (2.00 to 5.99)	4.69 (1.54 to 14.30)
Not White British		0.84 (0.41 to 1.73)	1.31 (0.70 to 2.47)	0.77 (0.44 to 1.35)	2.79 (0.82 to 9.48)
Left full-time education aged 17 or older		1.21 (0.73 to 1.99)	1.10 (0.72 to 1.68)	2.29 (1.55 to 3.40)	1.22 (0.51 to 2.92)
Lonely (6-item de Jong Gierveld)		1.79 (1.05 to 3.05)	1.71 (1.08 to 2.72)	1.65 (1.07 to 2.52)	5.41 (2.12 to 13.79)

For peer review only

Table 4. Odds ratios (95% confidence interval) from multi-variable adjusted regression analyses for the four final models: one for each type of resource use.

Four models →		(A) Hospital use	(B) Primary care use	(C) Other health care services	(D) Wash/Meals assistance
**Dependent variable: frequent internet use**		**0.98 (0.25 to 3.84)**	**1.15 (0.78 to 1.70)**	**1.72*** (1.33 to 2.23)**	**0.56 (0.12 to 2.55)**
Age bands	65-74 years	reference case	reference case	reference case	reference case
	75-84 years	1.50 (0.79 to 2.85)	1.35 (0.92 to 1.98)	1.08 (0.57 to 2.07)	3.44*** (1.69 to 6.97)
	85+ years	0.60 (0.15 to 2.44)	2.01 (0.54 to 7.41)	1.15 (0.45 to 2.97)	19.36*** (8.21 to 45.64)
Gender (male)		1.68* (1.01 to 2.81)	0.97 (0.68 to 1.39)	0.59*** (0.50 to 0.70)	0.69 (0.24 to 1.95)
Site (urban)		1.47* (1.07 to 2.04)	0.75 (0.37 to 1.54)	1.24 (0.84 to 1.84)	1.46 (0.60 to 3.52)
Season at start (autumn)		0.86 (0.71 to 1.05)	0.73* (0.56 to 0.95)	0.77 (0.57 to 1.04)	1.46 (0.81 to 2.63)
SF-12 mental score		1.01 (0.99 to 1.03)	0.96*** (0.95 to 0.98)	0.99 (0.94 to 1.04)	0.95 (0.91 to 1.00)
SF-12 physical score		0.94*** (0.92 to 0.96)	0.95** (0.93 to 0.98)	0.97* (0.95 to 1.00)	0.87*** (0.84 to 0.91)
Recent sudden illness		4.80*** (2.46 to 9.41)	1.89** (1.22 to 2.94)	2.27** (1.27 to 4.07)	1.42 (0.75 to 2.71)
Not White British		0.43** (0.25 to 0.75)	1.28 (0.51 to 3.18)	0.42** (0.24 to 0.72)	0.69 (0.16 to 2.95)
Left full-time education aged 17 or older		1.68*** (1.35 to 2.09)	1.23 (0.78 to 1.94)	2.72*** (2.19 to 3.39)	3.91*** (2.31 to 6.63)
Lonely (6-item de Jong Gierveld)		1.42 (0.80 to 2.51)	0.99 (0.64 to 1.54)	1.08 (0.56 to 2.06)	1.14 (0.54 to 2.39)

* p-value <0.05; ** p-value <0.01; *** p-value <0.001.

Discussion

Our results show that, in this relatively healthy older adult population, there was a strong and positive relationship between frequent internet use and use of any community-based health services such as physiotherapist, osteopath/ chiropractor, dentist, optician/optometrist, hearing clinic/audiologist, counsellor, smoking cessation service, chiropodist/podiatrist, and calls to the emergency services (see Appendix).

For peer review only

It is not possible to infer a causal relationship between frequent internet use and community health service use based on this analysis. The relationship could have arisen due to one of the following reasons: participants using the internet in order to research services that they wish to use; participants using services being influenced by other service users or other associated factors and thereby encouraged to use the internet. However, there could equally be no relationship at all, as correlation does not imply causation: those interested in and capable of using the internet might simply also be more aware of what services are on offer.

In addition to this, we did not observe disadvantages in terms of accessing primary or secondary health care in those who used the internet infrequently, although the study was not powered to detect such differences. We also did not observe a disadvantage in accessing informal assistance with washing and meals. No firm conclusions can be drawn, however, as we do not know from the study what the internet use entailed; for example, if participants used the internet to find out information about their health or local health care services. There are various initiatives under way to increase the online presence and activity of GP practices, and our results are consistent with preliminary suggestions that there is no cause for concern regarding increasing inequity of access for older people as a whole, though there may be smaller sub-groups within this population who are adversely affected. This is likely to be the case but we are not powered to look at this. It may also be that use of online GP services by younger or more technologically literate patients frees up time for receptionists to respond to older adults' telephone calls (25).

Limitations of this analysis are that the sample size is relatively small, and that resource use was binary, rather than counting the number of contacts that participants had (this was due to missing responses to sub-questions regarding the numbers of specific contacts). In addition, no causality can be inferred due to the nature of the study, and we do not have comprehensive information on the reasons for participants' internet use. We cannot speculate on how much of the internet use was specifically for looking up information on health, as opposed to for example keeping in touch with family and friends, or obtaining information on transport services or tradespeople, for example. The population that took part in this study has been compared to 2011 census data, and the study population was slightly younger, more likely to be owner-occupiers, and less likely to be in an ethnic minority than the census population (16) (17).

Implications

This is one of only a few studies that has investigated internet use alongside the use of other services. Our findings were exploratory and suggest the need for further research to better understand the relationships. In the future, in order to obtain more precise information on the nature of the relationship between

For peer review only

technology use and use of health or social care services, further detail could be asked regarding the purpose of internet use. Online technology changes very quickly, and this study offers a timely uptake on its use by older people living at home. Future work should aim to understand more regarding how older people use technology for their own healthcare both in terms of content and as a way to access information.

Contributorship Statement:

CSC, JR and SM designed the analysis, with important intellectual input from KK, JF, JM, SI, CG and KW. CSC cleaned the dataset with assistance from KK. CSC conducted the analysis and prepared the manuscript draft. All authors gave valuable input during the drafting process and approved the final manuscript.

References

1. Oliver D, Foot C, Humphries R. Making our health and social care systems fit for an ageing population. [Online].: The King's Fund; 2014 [cited July 2016. Available from: http://www.kingsfund.org.uk/publications/making-our-health-and-care-systems-fit-ageing-population.
2. Beswick AD, Rees K, Dieppe P, Ayis S, Gooberman-Hill R, Horwood J, et al. Complex interventions to improve physical function and maintain independent living in elderly people: a systematic review and meta-analysis. Lancet. 2008; 371: p. 725-735.
3. Slegers K, van Boxtel MPJ, Jolles J. Computer use in older adults: Determinants and the relationship with cognitive change over a 6 year episode. Computers in Human Behaviour. 2012; 28: p. 1-10.
4. Lambert SD, Loiselle CG. Health Information–Seeking Behavior. Qualitative Health Research. 2007;(8): p. 1006-1019.
5. International Longevity Centre. A New Journey to Health – health information seeking behaviour across the generations. [Online].: ILC; 2015 [cited July 2016. Available from: www.ilcuk.org.uk.
6. NHS England and Tinder Foundation. Health & Digital: Reducing inequalities, improving society - An evaluation of the Widening Digital Participation programme. [Online].; 2016 [cited July 2016. Available from: http://www.tinderfoundation.org/our-thinking/news/widening-digital-participation-health-report-launches-today.
7. Hertzog C, Kramer AF, Wilson RS, Lindenberger U. Cognitive Development Can the Functional Capacity of Older Adults Be Preserved and Enhanced? Psychological Science in the Public Interest. 2008; 9(1): p. 1-65.
8. Xavier AJ, d'Orsi E, de Oliveira CM, Orrell M, Demakakos P, Biddulph JP, et al. English Longitudinal Study of Aging: Can Internet/E-mail Use Reduce Cognitive Decline? Journals of Gerontology. Series A, Biological sciences and medical sciences. 2014; 69(9): p. 1117-1121.
9. Office for National Statistics (ONS). ONS. [Online].; 2016 [cited September 2016. Available from:

For peer review only

http://www.ons.gov.uk/businessindustryandtrade/itandinternetindustry/bulletins/internetusers/2016.

10. Choi N. Relationship between health service use and health information technology use among older adults: analysis of the US National Health Interview Survey. Journal of Medical Internet Research. 2011; 13(2): p. e33.

11. Choi NG, DiNitto DM. Internet use among older adults: association with health needs, psychological capital, and social capital. Journal of Medical Internet Research. 2013; 15(5): p. e97.

12. Elliot AJ, Mooney CJ, Douthit KZ, Lynch MF. Predictors of older adults' technology use and its relationship to depressive symptoms and well-being. The Journals of Gerontology Series B: Psychological Sciences and Social Sciences. 2014; 69(5): p. 667-677.

13. Damant J, Knapp M, Freddolino P, Lombard D. Effects of digital engagement on the quality of life of older people. Health & social care in the community. 2016.

14. Pew Research Center. Older Adults and Technology Use. [Online].; 2014 [cited September 2016. Available from: http://www.pewinternet.org/2014/04/03/older-adults-and-technology-use/.

15. Barnett K, Mercer SW, Norbury M, Watt G, Wyke S, Guthrie B. Epidemiology of multimorbidity and implications for health care, research, and medical education: a cross-sectional study. The Lancet. 2012; 380(9836): p. 37-43.

16. Walters K, Kharicha K, Goodman C, Handley M, Manthorpe J, Cattan M, et al. Promoting health and well-being for older people: a feasibility study of a new multi-dimensional approach in primary care. BMC Family Practice. Accepted.

17. Walters K, Kharicha K, Iliffe S, Goodman C, Handley M, Manthorpe J, et al. Promoting health and wellbeing for older people in primary care: a feasibility study. Lancet. 2013; 382(Special Issue S99).

18. Stuck AE, Elkuch P, Dapp U, Anders J, Iliffe S, Swift CGftPApsg. Feasibility and yield of a self-administered questionnaire for health risk appraisal in older people in three European countries. Age & Ageing. 2002; 31: p. 463-467.

19. Iliffe S, Kharicha K, Harari D, Swift C, Stuck AE. Health risk appraisal for older people in general practice using an expert system: a pilot study. Health and Social Care in the Community. 2005; 13: p. 21-29.

20. Harari D, Iliffe S, Kharicha K, Egger M, Gillman G, von Renteln-Kruse W, et al. Promotion of health in older people: an RCT of health risk appraisal in British general practice. Age & Ageing. 2008; 37(5): p. 565-571.

21. Manthorpe J, Kharicha K, Goodman C, Harari D, Swift C, Iliffe S. Smarter Working in Social and Health Care: Professional Perspectives on a New Technology for Risk Appraisal with Older People. British Journal of Social Work. 2010; 40(6): p. 1829-1846.

22. Iliffe S, Kharicha K, Harari D, Swift C, Goodman C, Manthorpe J. User involvement in the development of a health promotion technology for older people: findings from the SWISH project. Health and Social Care in the Community. 2010; 18(2): p. 147-159.

23. de Jong Gierveld J, van Tilburg T. De ingekorte schaal voor algemene, emotionele en sociale eenzaamheid [A shortened scale for overall, emotional and social loneliness]. Tijdschrift voor

For peer review only

Gerontologie en Geriatrie. 2008; 39: p. 4-15.

24. Ware JJ, Kosinski M, Keller SD. A 12-Item Short-Form Health Survey: construction of scales and preliminary tests of reliability and validity. Medical Care. 1996; 34(3): p. 220-233.

25. Lau R, Murray E, Stevenson F. Implementing Change to Improve Access in UK General Practice: A Mixed-methods Exploratory Case Study. In SAPC Annual Scientific Meeting; 2016; Dublin. p. Talk code 3D.1.

For peer review only

Appendix

A section of the Service use diary completed by participants at baseline, 3 and 6 months. The binary Y/N question for section D was a new variable generated by assigning a "Yes" for any participant who responded "Yes" to any sub-questions in that section.

ID:

Diary Two

We would like to know about any contact you have had with services, organisations or others about your health and well-being.

Please fill in the number of contacts you have had **during the last three months** for each question listed below. If you haven't had any contact with them, please fill in a 0 (zero).

**A.** Have you needed to go to **hospital in the last 3 months?**<br>Yes ☐ Complete 1-4 below<br>No ☐ Go to Section B	
1. How many nights have you spent in hospital as an inpatient?	
2. How many days have you been admitted to a day ward in hospital without staying overnight?	
3. How many times have you had an appointment with a doctor in the hospital or have you attended a clinic appointment?	
4. How many times have you been treated at Casualty or Accident and Emergency?	
**B.** Have you seen/spoken your **GP or community nurse in the last 3 months**?<br>Yes ☐ Complete 1-7 below<br>No ☐ Go to Section C	
1. How many times have you visited your GP at their surgery or health centre?	
2. How many times has a GP visited you at home?	
3. How many times have you spoken to a GP on the phone?	
4. How many times has a nurse visited you at home?	
5. How many times have you visited a nurse at the surgery or health centre?	
6. How many times have you spoken to a nurse on the phone?	
7. How many times have you called NHS Direct?	

ID:

**C.** Have you contacted/been to **any other health care services in the last 3 months?** Yes ☐ Complete 1-10 below; No ☐ Go to Section D	
1. How many times have you seen a physiotherapist? (please indicate if NHS or private) ………………………………………..	
2. How many times have you seen an osteopath or chiropractor (please indicate if NHS or private)?………………………………	
3. How many times have you seen a dentist (please indicate if NHS or private)?……………………………………………………	
4. How many times have you seen someone about your sight (eg optician / optometrist?)	
5. How many times have you seen someone about your hearing (eg an audiologist/ or an ear/hearing clinic?)	
6. How many times have you been to a counselling service?	
7. How many times have you been to a smoking cessation service?	
8. How many times have you been to a chiropodist or podiatrist (foot) clinic?	
9. How many times have you contacted any emergency services (Police, Ambulance, Fire)? Please give details …………………………………………………………………	
10. How many times have you used *another* health service contact? e.g. falls prevention service. Please give details …………………………………………………………………………	

D. Personal care
*Please think about an average or typical week during the **last 3 months***

Have you had any **paid** help (including both help arranged or paid for by social services and help arranged/paid for privately) with:

1. Washing, dressing or having a bath/shower

Yes ☐ No ☐ If yes, how many times *per week*? …………..

2. Cooking/preparing meals or shopping

Yes ☐ No ☐ If yes, how many times *per week*? ……………

3. Meals on wheels or other meal delivery service

Yes ☐ No ☐ If yes, how many times *per week*? …………..

4. Other (eg laundry, sitting service): please give details and number of times *per*

ID:

Have you had any **unpaid** help e.g. from family or friends, with:

5. Washing, dressing or having a bath/shower

Yes ☐ No ☐ If yes, how many times *per week*? ……………

6. Cooking/preparing meals or shopping

Yes ☐ No ☐ If yes, how many times *per week*? …………...

7. Other (eg laundry, sitting service): please give details and number of times *per week*?

…………………………………………………………………………………………...

For peer review only

BMJ Open

Exploring the relationship between frequent internet use and health and social care resource use in a community-based cohort of older adults

Journal:	*BMJ Open*
Manuscript ID	bmjopen-2017-015839.R1
Article Type:	Research
Date Submitted by the Author:	31-Mar-2017
Complete List of Authors:	Clarke, Caroline; University College London, Research Department of Primary Care and Population Health<br>Round, Jeff; University of Bristol School of Social and Community Medicine<br>Morris, Stephen; University College London, Department of Applied Health Research<br>Kharicha, Kalpa; University College London, Primary Care and Population Health<br>Ford, John; University of East Anglia, Public Health<br>Manthorpe, Jill; King's College London, Social Care Workforce Research Unit<br>Iliffe, Steve; University College London, Primary Care and Population Sciences<br>Goodman, Claire; University of Hertfordshire, Centre for Research in Primary and Community Care<br>Walters, Kate; University College London, Primary Care and Population Health
**Primary Subject Heading**:	General practice / Family practice
Secondary Subject Heading:	Health services research, Geriatric medicine
Keywords:	Older adults, Health service resource use, Internet use, Panel data, Logistic regression, PRIMARY CARE

SCHOLARONE™
Manuscripts

Exploring the relationship between frequent internet use and health and social care resource use in a community-based cohort of older adults

Caroline S. Clarke[1], Jeff Round[2], Stephen Morris[3], Kalpa Kharicha[1], John Ford[4], Jill Manthorpe[5], Steve Iliffe[1]*, Claire Goodman[6], Kate Walters[1]

Affiliations:

[1] UCL Research Dept. of Primary Care & Population Health, UCL Medical School, Upper 3rd Floor, Royal Free Campus, Rowland Hill St, London, NW3 2PF, UK

[2] School of Social and Community Medicine, University of Bristol, Canynge Hall, 39 Whatley Road, Bristol, BS8 2PS, UK

[3] Department of Applied Health Research, University College London (UCL), 1-19 Torrington Place, London, WC1E 7HB, UK

[4] Department of Public Health and Primary Care, Norwich Medical School, University of East Anglia, Chancellors Drive, Norwich, NR4 7TJ, UK

[5] Social Care Workforce Research Unit, King's College London, Strand, London, WC2 4LF, UK

[6] Centre for Research in Primary and Community Care, University of Hertfordshire, AL10 9AB, UK

* Emeritus

Corresponding author:

Email: caroline.clarke@ucl.ac.uk

Tel.: +44 (0)20 7794 0500 ext. 36770

For peer review only - http://bmjopen.bmj.com/site/about/guidelines.xhtml

ABSTRACT

Objectives

Given many countries' ageing populations, policymakers must consider how to mitigate or reduce health problems associated with old age, within budgetary constraints. Evidence of use of digital technology in delaying the onset of illness and reducing healthcare service use is mixed, with no clear consensus as yet. Our aim was to investigate the relationship between frequent internet use and patterns of health or social care resource use in primary care attendees who took part in a study to improve the health of older adults.

Methods

Participants recruited from primary care, aged over 65 and living in semi-rural or urban areas in the south of England, were followed up at 3 and 6 months after completing a comprehensive questionnaire with personalised feedback on their health and well-being. We performed logistic regression analyses to investigate relationships between frequent internet use and patterns of service use, controlling for confounding factors, and clustering by GP practice. Four categories of service use data were gathered: use of primary NHS care; secondary NHS care; other community health and social care services; and assistance with washing, shopping and meals.

Results

Our results show, in this relatively healthy population, a positive relationship (odds ratio 1.72; 95% CI 1.33 to 2.23) between frequent internet use and use of any other community-based health services (physiotherapist, osteopath/chiropractor, dentist, optician/optometrist, counselling service, smoking cessation service, chiropodist/podiatrist, emergency services, other non-specific health services), and no relationship with the other types of care. No causal relationship can be postulated due to the study's design.

Conclusions

No observed relationship between frequent internet use and primary or secondary care use was found, suggesting that older adults without internet access are not disadvantaged regarding health care utilisation. Further research should explore how older people use the internet to access healthcare, and the impact on health.

Key words: Older adults, health service resource use, internet use, panel data, logistic regression, primary care.

For peer review only - http://bmjopen.bmj.com/site/about/guidelines.xhtml

ARTICLE SUMMARY

Strengths and limitations of this study

Strengths:

- Timely study providing an update on older adults' use of the internet at home and on their use of health and other care services.
- Lack of bias: the question on internet use was one of many, so participants will not have attached much weight to it and this should minimise any reporting bias.

Limitations:

- We cannot speculate on how much of the internet use was specifically for looking up information on health or accessing health-related services, as opposed to general correspondence, or looking up information on any other non-health services, for example.
- Causality cannot be inferred.
- Small study size (n=454)

Funding: The WISH study was funded by the Medical Research Council (MRC) LLHW G1001822/1. The MRC had no role in the design, collection, analysis, or interpretation of data; in the writing of this manuscript; or in the decision to submit the manuscript for publication. Ethical approval for the WISH study was granted by London-East Research Ethics Committee (reference 11/LO/1814) which included permissions to conduct the analysis reported in this paper. The corresponding author for this analysis was not involved in the WISH study and received no specific grant from any funding agency in the public, commercial or not-for-profit sectors.

Conflict of Interest: All authors have completed the ICMJE uniform disclosure form at www.icmje.org/coi_disclosure.pdf and declare: no support from any organisation for the submitted work; no financial relationships with any organisations that might have an interest in the submitted work in the previous three years; no other relationships or activities that could appear to have influenced the submitted work.

Data sharing statement: no additional data available.

For peer review only

INTRODUCTION

Life expectancy is rising worldwide, and much research seeks to find ways of improving older people's health and well-being (1). Work has been undertaken by various groups regarding complex interventions designed to alter behaviour to improve health and well-being, enabling older adults to maintain their independence and good health for longer, however there is no clear consensus on the best approaches(2) (3) (4) (5). It has been argued that the use of technology by older people could help in maintaining health and well-being and/or assist in managing or reducing health-related resource use(6) (7); similarly, other work has suggested that older adults might be disadvantaged if they do not use information and communications technology regularly(8).

There is significant use of the internet by older people, particularly by those in their 60s and 70s, but it is not universal and decreases with age(9). Research on how older people's use of the internet might influence the way they seek help/use healthcare and other resources is still in its infancy(10)(11)(12)(13). There seem to be differences between how younger and older people use the internet; for example, older adults who use the internet seem to use it primarily for email, whereas a large proportion of younger people use social media sites, both for information and for socialising(14). As the majority of older people have multi-morbidities with increasingly complex health and other needs(15), this might influence their use of the internet in relation to their health, as well as there being differences in digital and health literacy in comparison to younger sections of the population.

In the UK, some GP practices offer online services to patients, including appointment booking systems, and even online access to patients' own primary care records, although this latter example is not yet widely established. Also, health care providers are now assessed and ranked, and patients' opinions regarding services can be found online on the NHS Choices website. It is not yet clear what the uptake and impact of these various NHS online services are across age groups and among other sections of society, but it is conceivable that not using the internet might hinder use of these services and therefore access to health care.

Participants in the WISH (Well-being Interventions for Social and Health needs) study (16) were community-dwelling older adults recruited from English primary care settings in the London Borough of Ealing (urban) and Hertfordshire (semi-rural) and they were sent the Multi-dimensional Risk Appraisal in Older people (MRA-O) as a postal questionnaire. The MRA-O is an extension of the Health Risk Appraisal in Older people (HRA-O) system (17) (18) (19), including domains identified as having an impact on health and well-being in

For peer review only - http://bmjopen.bmj.com/site/about/guidelines.xhtml

later life during the Smarter Working in Social and Health Care (SWISH) project (20) (21). Participants were asked questions covering a broad range of health, lifestyle, social and environmental domains, including questions on their use of the internet. The resource use data included information on a wide range of services, both public and privately funded, and data on use of the internet, meaning that this dataset could enable us to explore the relationship between internet use and resource use, while considering various possible confounders and adjusting for important covariates.

The aim of this study was to examine the relationship between frequent internet use and different types of health and social care resource use, and to consider whether differences in internet use raise concerns about equity of access and use of care services by older adults.

METHODS

The methods used in this analysis are compliant with the STROBE guidelines for observational cohort studies(22).

Design: Cohort study

Participants: A randomly sampled cohort of community-dwelling older adult participants aged 65 years and over from five general practices in two diverse regions of southern England, recruited in 2012 and followed up for 6 months as part of the WISH study (16).

Data collection: Potential participants were sent letters by their GPs on behalf of the study group, and 526 of the 1,550 contacted in this way responded. Of these, 454 returned the M-RAO. The data collected included physical and mental well-being, functional ability, lifestyle and diet, personal characteristics, loneliness and social networks, use of health care and social resources, and internet and mobile phone use. Further detail regarding the WISH study recruitment and data collection procedures are described elsewhere (16).

Measurements

Resource use

The WISH study measured resource use across a range of services, including primary and secondary healthcare, informal and other community health care, and support from informal or family carers or social care services. These were captured in this analysis as four individual binary resource use variables, where

For peer review only - http://bmjopen.bmj.com/site/about/guidelines.xhtml

For peer review only

"yes" meant that one or more of the difference types of contact listed below had occurred within the last three months:

A. Secondary care: hospital attendance (A&E, inpatient, outpatient)
B. Primary care: GP/community nurse consultation (by phone, face-to-face, a home visit, or a call to NHS Direct)
C. Other health care services (either NHS or private): physiotherapist, osteopath/chiropractor, dentist, optician/optometrist, hearing clinic/audiologist, counsellor, smoking cessation service, chiropodist/podiatrist, emergency services (police, ambulance, fire)
D. Wash/meals: any paid or unpaid help (e.g. from family member or social care services) with washing, dressing, having a bath/shower, cooking/preparing meals, shopping, or meal delivery service. The overall binary variable here returns a "yes" if any paid or unpaid help was reported.

Participants who responded 'Yes' were also asked sub-questions in each case, regarding how many contacts they had had with different services, e.g. how many nights the participant stayed in hospital, how many times they spoke with the general practice nurse on the phone. The complete list of questions can be found in the Appendix. The principal binary questions for the resource variables were used in the analysis instead of counting the numbers of contacts due to high levels of missing data in the sub-questions.

Internet use

The internet use question offered four possible answers: often (most days); sometimes (1-3 days a week); occasionally (less than once a week); and never. For the purposes of this analysis, it was dichotomised as "often/sometimes" (frequently) vs. "occasionally/never" (infrequently) as the numbers of responses across the four groups were too small to allow meaningful analysis as a four-category variable.

Covariates

We considered a wide range of patient characteristics for the analyses, including: GP practice location type (urban or semi-rural), season of study entry (summer or autumn), sex, age (in bands: 65-74 years, 75-84 years, 85+ years), ethnicity (White British or other), loneliness status (scoring 0-1 or 2-6 on the de Jong Gierveld 6-item short scale(23) corresponds to "not lonely" or "lonely", respectively), social isolation status (scoring below 12 on the Lubben Social Network Scale corresponds to "socially isolated"), binary response to "Do you feel lonely much of the time?", Short Form SF-12(24) mental health component summary score (MCS) and physical health component summary score (PCS), occurrence of a recent sudden illness in the 3 months before baseline, age at which left full-time education (before or after 17 years of age), and receipt of pension (state pension only vs. other). GP practice location type was included because patterns of health care resource use necessarily vary according to population and practice density. Season of study entry was

For peer review only - http://bmjopen.bmj.com/site/about/guidelines.xhtml

included as there is evidence that use of health care services is seasonal(25), and the loneliness and social isolation variables were included as there has been some research suggesting that, particularly in older adults, use of health care services can sometimes be a substitute for social contact(26)(27). The SF-12 was included as a short quality-of-life measure, and this measure is reported, as is usual, as its two components: the MCS and the PCS(24). Pension type and the age at which the participant left full-time education were included as proxy measures for socioeconomic status(28)(29). The simplicity of the ethnic group division chosen was due to low participant numbers in any non-White-British group, particularly in the semi-rural practices.

Analysis

We undertook panel logistic regression for each of the four dichotomous dependent outcome variables on service use, with the GP surgery contributing random effects. This was included as certain variables could be affected in some way by the GP practice's local policies or working practices, meaning that including these possible effects as random was the most appropriate choice. The covariates for the final multivariate regression models were chosen using the common model-selection criteria, the Akaike information criterion (AIC) and the Bayesian information criterion (BIC). Interactions between certain variables were also tested. We report odds ratios and 95% confidence intervals to investigate the relationship between frequent internet use and different types of resource use, controlling for patient characteristics described above. The age group variable was included as a factor variable to remove the assumption of a linear effect with age. Its joint significance was also tested using the chi-squared test. The data were set in Stata as panel data using the patient ID code as the panel variable, and the number of months' follow-up was set as the time variable (0, 3 and 6 months), although exclusion of the time variable when setting the data led to no difference in the regression results.

Missing data

Demographic data were completed by all 454 participants who returned the M-RAO, except for 7 missing responses to the ethnicity question. Other questions and sub-questions were not always completed. We used complete case analysis for the four panel regression models and have not imputed any missing data. Numbers of missing data in each case are detailed in the tables below as required, with the largest proportion of missing data at baseline being 11% (50/454) in the de Jong Gierveld loneliness variable. Most variables in these analyses had much lower proportions of missing data (~2%). With such low rates of missing data, it was decided that undertaking multiple imputation to estimate new values would not be an efficient use of time. At later timepoints there were some drop-outs, leading to 89% retention at the 3-month timepoint and 77% retention at 6 months.

For peer review only - http://bmjopen.bmj.com/site/about/guidelines.xhtml

For peer review only

RESULTS

Sample characteristics

Participants' use of the internet was asked as a four-category question: often, i.e. most days (44%); sometimes, i.e. 1-3 days a week (11%); occasionally, i.e. less than once a week (8%); and never (37%), and this was dichotomised as frequently (55%) and infrequently (45%). Patient characteristics for those covariates used in the final models, split by internet use, are given in Table 1. There was a large amount of missing data in the sub-questions regarding numbers of each specific type of contact in each of the four resource use types, with between 3.4% and 48.1% of those who responded "Yes" to the principal question failing to then state any numbers of contacts. The amount of missing data in the four principal binary service use variables was much smaller. The total number of participants in the WISH study at baseline was 454, dropping to 405 (89% retention) at the 3-month timepoint and 348 (77% retention) at 6 months, and only 4% or fewer participants who responded at each timepoint were excluded from the complete case analyses on the basis of missing resource use data (see Table 2).

Table 1. Characteristics of participants, split according to whether or not they used the internet frequently (complete cases only).

Covariates		Using internet infrequently, n=198	Using internet frequently, n=247	Overall n=454
Site (semi-rural; other option was urban)		63.6%	62.4%	62.3%
Season at start (autumn; other option was summer)		47.5%	48.2%	47.6%
Gender (female)		58.6%	48.6%	52.9%
Age bands	65-74 years	44.4%	73.7%	59.9%
	75-84 years	42.4%	24.3%	33.3%
	85+ years	13.1%	2.0%	6.8%
White British (7 missing)		84.4%	87.4%	86.1%
Lonely (6-item de Jong Gierveld score) (50 missing)		38.7%	31.2%	34.9%
SF-12 mental score (mean, SD) (42 missing)		52.6, 8.2	53.7, 8.9	53.2, 8.6
SF-12 physical score (mean, SD) (42 missing)		40.6, 12.7	46.5, 11.7	43.9, 12.5
Recent sudden illness (12 missing)		18.2%	15.7%	17.0%
Left FT education before 17 years of age (3 missing)		74.0%	49.6%	60.8%

For peer review only - http://bmjopen.bmj.com/site/about/guidelines.xhtml

Table 2. Complete cases of yes and no responses for the four binary resource use variables.

Resource use variable	Baseline n=454	3 months n=405	6 months n=348
(A) Secondary care – Yes	37%	40%	35%
– No	63%	60%	65%
	10 missing	2 missing	14 missing
(B) Primary care – Yes	74%	75%	71%
– No	26%	25%	29%
	11 missing	6 missing	15 missing
(C) Other health care – Yes	54%	57%	50%
– No	46%	43%	50%
	11 missing	3 missing	13 missing
(D) Wash/Meals – Yes	14%	18%	15%
– No	86%	82%	85%
	8 missing	2 missing	12 missing

Univariable unadjusted analyses

Shown in Table 3 are the raw unadjusted relationships between each of the covariates included as confounders in the final multi-variable models and each binary service use variable. These results show the relationship between each resource use variable and each covariate, with no controlling for any other covariate.

Multi-variable adjusted analyses

Models with controlling variables included were constructed using the AIC and BIC, and gave an improved fit to the data compared to the univariable models. The controlling variables included were: age, sex, site, season at start, SF-12 mental and physical component scores, having had a recent sudden illness, ethnicity, age at which left full-time education, and de Jong Gierveld loneliness status. Interactions between pension and internet use, between age at which left full-time education and internet use, and between binary social isolation variable derived from the Lubben Social Network Scale and binary response to "Do you feel lonely much of the time", were tested, but did not improve the model fit for any of the four regressions and so were not included. The multi-variable models' results are shown in Table 4 and outlined here below.

(A) Hospital use

When controlling for age, sex, site, season at start, SF-12 mental and physical component scores, having had a recent sudden illness, ethnicity, age at which left full-time education, and loneliness, there was no

For peer review only - http://bmjopen.bmj.com/site/about/guidelines.xhtml

observed association between hospital use and frequent internet use (OR 0.98, 95% CI 0.25 to 3.84) (see Table 4).

(B) Primary care

Use of primary care services, controlling for all the same variables, was also not associated with frequent internet use (OR 1.15, 95% CI 0.78 to 1.70) (see Table 4).

(C) Other healthcare

Frequent internet use was, however, positively associated with use of other health care services (e.g. optician, dentist, physiotherapist, etc.), when controlling for all the same variables (OR 1.72, 95% CI 1.33 to 2.23) (see Table 4).

(D) Washing/meals assistance

Of those participants who stated that they were using assistance of this nature, approximately a quarter were paying for these services. Receipt of assistance (paid or unpaid) for washing, cooking and similar tasks was not associated with frequent internet use, when controlling for all the same variables, (OR 0.56, 95% CI 0.12 to 2.55) (see Table 4).

Table 3. Odds ratios (95% confidence interval) from univariable unadjusted analyses of each individual covariate and its relationship to each resource use variable.

Independent variables		(A) Hospital use	(B) Primary care use	(C) Other health care services	(D) Wash/Meals assistance
Frequent internet use		0.76<br>(0.47 to 1.25)	0.79<br>(0.52 to 1.20)	1.42<br>(0.97 to 2.09)	0.12***<br>(0.05 to 0.29)
Age bands	65-74 years	reference case	reference case	reference case	reference case
	75-84 years	1.44<br>(0.85 to 2.44)	1.54<br>(0.98 to 2.42)	0.94<br>(0.62 to 1.42)	7.36***<br>(2.98 to 18.19)
	85+ years	1.55<br>(0.59 to 4.08)	4.76***<br>(1.70 to 13.36)	1.50<br>(0.68 to 3.34)	176.38***<br>(33.87 to 918.41)
Gender (male)		1.13<br>(0.69 to 1.84)	0.95<br>(0.62 to 1.43)	0.57**<br>(0.39 to 0.83)	0.60<br>(0.25 to 1.41)
Site (urban)		1.35<br>(0.82 to 2.23)	0.95<br>(0.62 to 1.46)	1.40<br>(0.94 to 2.08)	2.76*<br>(1.15 to 6.63)
Season at start (autumn)		0.71<br>(0.44 to 1.16)	0.71<br>(0.47 to 1.07)	0.84<br>(0.57 to 1.24)	2.26<br>(0.95 to 5.37)
SF-12 mental score		0.97<br>(0.94 to 1.00)	0.96**<br>(0.93 to 0.98)	0.97**<br>(0.94 to 0.99)	0.94*<br>(0.89 to 0.99)
SF-12 physical score		0.94***<br>(0.92 to 0.96)	0.95***<br>(0.93 to 0.97)	0.98**<br>(0.96 to 0.99)	0.85***<br>(0.82 to 0.89)

For peer review only

For peer review only - http://bmjopen.bmj.com/site/about/guidelines.xhtml

Recent sudden illness	5.07***<br>(2.68 to 9.59)	3.40***<br>(1.79 to 6.46)	3.46***<br>(2.00 to 5.99)	4.69**<br>(1.54 to 14.30)
Not White British	0.84<br>(0.41 to 1.73)	1.31<br>(0.70 to 2.47)	0.77<br>(0.44 to 1.35)	2.79<br>(0.82 to 9.48)
Left full-time education aged 17 or older	1.21<br>(0.73 to 1.99)	1.10<br>(0.72 to 1.68)	2.29***<br>(1.55 to 3.40)	1.22<br>(0.51 to 2.92)
Lonely (6-item de Jong Gierveld)	1.79*<br>(1.05 to 3.05)	1.71*<br>(1.08 to 2.72)	1.65*<br>(1.07 to 2.52)	5.41***<br>(2.12 to 13.79)

* p-value <0.05; ** p-value <0.01; *** p-value <0.001.

For peer review only

Table 4. Odds ratios (95% confidence interval) from multi-variable adjusted regression analyses for the four final models: one for each type of resource use.

**Four models →**		**(A) Hospital use**	**(B) Primary care use**	**(C) Other health care services**	**(D) Wash/Meals assistance**
**Dependent variable: frequent internet use**		**0.98**<br>**(0.25 to 3.84)**	**1.15**<br>**(0.78 to 1.70)**	**1.72***<br>**(1.33 to 2.23)**	**0.56**<br>**(0.12 to 2.55)**
Age bands	65-74 years	reference case	reference case	reference case	reference case
	75-84 years	1.50<br>(0.79 to 2.85)	1.35<br>(0.92 to 1.98)	1.08<br>(0.57 to 2.07)	3.44***<br>(1.69 to 6.97)
	85+ years	0.60<br>(0.15 to 2.44)	2.01<br>(0.54 to 7.41)	1.15<br>(0.45 to 2.97)	19.36***<br>(8.21 to 45.64)
Gender (male)		1.68*<br>(1.01 to 2.81)	0.97<br>(0.68 to 1.39)	0.59***<br>(0.50 to 0.70)	0.69<br>(0.24 to 1.95)
Site (urban)		1.47*<br>(1.07 to 2.04)	0.75<br>(0.37 to 1.54)	1.24<br>(0.84 to 1.84)	1.46<br>(0.60 to 3.52)
Season at start (autumn)		0.86<br>(0.71 to 1.05)	0.73*<br>(0.56 to 0.95)	0.77<br>(0.57 to 1.04)	1.46<br>(0.81 to 2.63)
SF-12 mental score		1.01<br>(0.99 to 1.03)	0.96***<br>(0.95 to 0.98)	0.99<br>(0.94 to 1.04)	0.95<br>(0.91 to 1.00)
SF-12 physical score		0.94***<br>(0.92 to 0.96)	0.95**<br>(0.93 to 0.98)	0.97*<br>(0.95 to 1.00)	0.87***<br>(0.84 to 0.91)
Recent sudden illness		4.80***<br>(2.46 to 9.41)	1.89**<br>(1.22 to 2.94)	2.27**<br>(1.27 to 4.07)	1.42<br>(0.75 to 2.71)
Not White British		0.43**<br>(0.25 to 0.75)	1.28<br>(0.51 to 3.18)	0.42**<br>(0.24 to 0.72)	0.69<br>(0.16 to 2.95)
Left full-time education aged 17 or older		1.68***<br>(1.35 to 2.09)	1.23<br>(0.78 to 1.94)	2.72***<br>(2.19 to 3.39)	3.91***<br>(2.31 to 6.63)
Lonely (6-item de Jong Gierveld)		1.42<br>(0.80 to 2.51)	0.99<br>(0.64 to 1.54)	1.08<br>(0.56 to 2.06)	1.14<br>(0.54 to 2.39)

* p-value <0.05; ** p-value <0.01; *** p-value <0.001.

For peer review only - http://bmjopen.bmj.com/site/about/guidelines.xhtml

DISCUSSION

Our results show that, in this relatively healthy older adult population, there was a strong and positive relationship between frequent internet use and use of any community-based health services such as physiotherapist, osteopath/chiropractor, dentist, optician/optometrist, hearing clinic/audiologist, counsellor, smoking cessation service, chiropodist/podiatrist, and calls to the emergency services (see Appendix). Use of the internet could be implicated in a person's ability to find any of these community-based services, except perhaps the emergency services. It is not possible to infer a causal relationship between frequent internet use and community health service use based on this analysis. The relationship could have arisen due to one of the following reasons: participants using the internet in order to research services that they wish to use; or participants using services being influenced by other service users or other associated factors and thereby encouraged to use the internet. However, there could equally be no relationship at all, as correlation does not imply causation: those interested in and capable of using the internet might simply also be more aware of what services are on offer.

We did not observe disadvantages in terms of accessing primary or secondary health care in those who used the internet infrequently, although the study was not powered to detect such differences. We also did not observe a disadvantage in accessing informal assistance with washing and meals. This is perhaps surprising as needing assistance with washing and meals suggests significant impairment in functioning, which might also impact on internet use. No firm conclusions can be drawn, however, as we do not know from the study what the internet use entailed; for example, if participants used the internet to find out information about their health or local health care services or otherwise.

There are a number of factors that can contribute to the digital divide between older and younger age groups. These can include a lack of infrastructure, i.e. lack of access to broadband and/or wi-fi, as well as individual difficulties with learning how to use the internet for those who are acquiring these skills in later life(30). Research on how older people's use of the internet might influence the way they seek help or use healthcare and other resources is still in its infancy(10)(11)(12)(13), and it is thought that, besides differences in digital and health literacy in comparison to younger sections of the population, older people's complex co-morbidities and other needs(15) might also influence their use of the internet in relation to their health.

Statistics published by the ONS(9) state that levels of internet use are growing, and the proportion of adults who had used the internet either never or longer than 3 months ago had decreased by 13.3 percentage

For peer review only - http://bmjopen.bmj.com/site/about/guidelines.xhtml

points since 2011. Women over the age of 75 have undergone the largest rise in 'recent' (i.e. having used the internet in the last 3 months) internet use since 2011, although it remains that less than a third of this group (32.6%) were recent users in 2016. People aged 75 years and over consistently have the lowest internet usage rates, in agreement with our observations, but these rates are increasing: from 19.9% of this age group in 2011, to 33.0% in 2015, and 38.7% in 2016 (9). These figures suggest that the digital divide between younger and older age groups might be diminishing in terms of a simple measure of internet use.

There are various initiatives under way to increase the online presence and activity of GP practices(31), but some concerns have been raised that this might disadvantage those who use the internet less frequently, for example some older adults, particularly women aged over 75, or other disadvantaged groups such as those with disabilities(9). On the other hand, it has also been postulated that use of online GP services by younger or more technologically literate patients frees up time for receptionists to respond to older adults' telephone calls (32). Our results are consistent with preliminary suggestions that there might be no cause for concern regarding increasing inequity of access for older people as a whole, though there may be smaller sub-groups within this population who are adversely affected. This present study lacked sufficient power to confirm or refute this, and our patient group was a relatively healthy group, recruited via primary care.

Limitations of this analysis are that the sample size is relatively small, and that resource use was binary, rather than counting the number of contacts that participants had (this was due to missing responses to sub-questions regarding the numbers of specific contacts). In addition, no causality can be inferred due to the nature of the study, and we do not have comprehensive information on the reasons for participants' internet use. We cannot speculate on how much of their internet use was specifically for looking up information on health, as opposed to for example keeping in touch with family and friends, or obtaining information on transport services or tradespeople, for example. The population that took part in this study has been compared to 2011 census data, and the study population was slightly younger, more likely to be owner-occupiers, and less likely to be in an ethnic minority than the census population(16).

Implications

This is one of only a few studies that has investigated internet use alongside the use of other services. Our findings were exploratory and suggest the need for further research to better understand the relationships. In the future, in order to obtain more precise information on the nature of the relationship between technology use and use of health or social care services, further detail could be asked regarding the purpose of internet use, actions taken as a result of internet access, and what type of device is used to access the internet. Online technology changes very quickly, and this study offers a timely update on its use by older

For peer review only - http://bmjopen.bmj.com/site/about/guidelines.xhtml

people living at home. Future work should aim more to understand how older people use technology for their own healthcare both in terms of content and as a way to access information. The use of the internet by older people in long-term care facilities and in hospitals remains under-explored.

Contributorship Statement:

CSC, JR and SM designed the analysis, with important intellectual input from KK, JF, JM, SI, CG and KW. CSC cleaned the dataset with assistance from KK. CSC conducted the analysis and prepared the manuscript draft. All authors gave valuable input during the drafting process and approved the final manuscript.

For peer review only

REFERENCES

1. Oliver D, Foot C, Humphries R. Making our health and social care systems fit for an ageing population. [Online].: The King's Fund; 2014 [cited July 2016. Available from: http://www.kingsfund.org.uk/publications/making-our-health-and-care-systems-fit-ageing-population.

2. Beswick AD, Rees K, Dieppe P, Ayis S, Gooberman-Hill R, Horwood J, et al. Complex interventions to improve physical function and maintain independent living in elderly people: a systematic review and meta-analysis. Lancet. 2008; 371: p. 725-735.

3. Slegers K, van Boxtel MPJ, Jolles J. Computer use in older adults: Determinants and the relationship with cognitive change over a 6 year episode. Computers in Human Behaviour. 2012; 28: p. 1-10.

4. Lambert SD, Loiselle CG. Health Information–Seeking Behavior. Qualitative Health Research. 2007;(8): p. 1006-1019.

5. International Longevity Centre. A New Journey to Health – health information seeking behaviour across the generations. [Online].: ILC; 2015 [cited July 2016. Available from: www.ilcuk.org.uk.

6. NHS England and Tinder Foundation. Health & Digital: Reducing inequalities, improving society - An evaluation of the Widening Digital Participation programme. [Online].; 2016 [cited July 2016. Available from: http://www.tinderfoundation.org/our-thinking/news/widening-digital-participation-health-report-launches-today.

7. Hertzog C, Kramer AF, Wilson RS, Lindenberger U. Cognitive Development Can the Functional Capacity of Older Adults Be Preserved and Enhanced? Psychological Science in the Public Interest. 2008; 9(1): p. 1-65.

8. Xavier AJ, d'Orsi E, de Oliveira CM, Orrell M, Demakakos P, Biddulph JP, et al. English Longitudinal Study of Aging: Can Internet/E-mail Use Reduce Cognitive Decline? Journals of Gerontology. Series A, Biological sciences and medical sciences. 2014; 69(9): p. 1117-1121.

9. Office for National Statistics (ONS). ONS. [Online].; 2016 [cited September 2016. Available from: http://www.ons.gov.uk/businessindustryandtrade/itandinternetindustry/bulletins/internetusers/2016.

10. Choi N. Relationship between health service use and health information technology use among older

For peer review only - http://bmjopen.bmj.com/site/about/guidelines.xhtml

adults: analysis of the US National Health Interview Survey. Journal of Medical Internet Research. 2011; 13(2): p. e33.

11. Choi NG, DiNitto DM. Internet use among older adults: association with health needs, psychological capital, and social capital. Journal of Medical Internet Research. 2013; 15(5): p. e97.
12. Elliot AJ, Mooney CJ, Douthit KZ, Lynch MF. Predictors of older adults' technology use and its relationship to depressive symptoms and well-being. The Journals of Gerontology Series B: Psychological Sciences and Social Sciences. 2014; 69(5): p. 667-677.
13. Damant J, Knapp M, Freddolino P, Lombard D. Effects of digital engagement on the quality of life of older people. Health & social care in the community. 2016.
14. Pew Research Center. Older Adults and Technology Use. [Online].; 2014 [cited September 2016. Available from: http://www.pewinternet.org/2014/04/03/older-adults-and-technology-use/.
15. Barnett K, Mercer SW, Norbury M, Watt G, Wyke S, Guthrie B. Epidemiology of multimorbidity and implications for health care, research, and medical education: a cross-sectional study. The Lancet. 2012; 380(9836): p. 37-43.
16. Walters K, Kharicha K, Goodman C, Handley M, Manthorpe J, Cattan M, et al. Promoting independence, health and well-being for older people: a feasibility study of computer-aided health and social risk appraisal system in primary care. BMC Family Practice. 2017; 18(47).
17. Stuck AE, Elkuch P, Dapp U, Anders J, Iliffe S, Swift CGftPApsg. Feasibility and yield of a self-administered questionnaire for health risk appraisal in older people in three European countries. Age & Ageing. 2002; 31: p. 463-467.
18. Iliffe S, Kharicha K, Harari D, Swift C, Stuck AE. Health risk appraisal for older people in general practice using an expert system: a pilot study. Health and Social Care in the Community. 2005; 13: p. 21-29.
19. Harari D, Iliffe S, Kharicha K, Egger M, Gillman G, von Renteln-Kruse W, et al. Promotion of health in older people: an RCT of health risk appraisal in British general practice. Age & Ageing. 2008; 37(5): p. 565-571.
20. Manthorpe J, Kharicha K, Goodman C, Harari D, Swift C, Iliffe S. Smarter Working in Social and Health Care: Professional Perspectives on a New Technology for Risk Appraisal with Older People. British Journal of Social Work. 2010; 40(6): p. 1829-1846.
21. Iliffe S, Kharicha K, Harari D, Swift C, Goodman C, Manthorpe J. User involvement in the development of a health promotion technology for older people: findings from the SWISH project. Health and Social Care in the Community. 2010; 18(2): p. 147-159.
22. von Elm E, Altman DG, Egger M, Pocock SJ, Gøtzsche PC, Vandenbroucke JP, et al. The Strengthening the Reporting of Observational Studies in Epidemiology (STROBE) statement: guidelines for reporting observational studies. PLoS Medicine. 2007 Oct 16; 4(10): p. e296.
23. de Jong Gierveld J, van Tilburg T. De ingekorte schaal voor algemene, emotionele en sociale eenzaamheid [A shortened scale for overall, emotional and social loneliness]. Tijdschrift voor Gerontologie en Geriatrie. 2008; 39: p. 4-15.

For peer review only - http://bmjopen.bmj.com/site/about/guidelines.xhtml

24. Ware JJ, Kosinski M, Keller SD. A 12-Item Short-Form Health Survey: construction of scales and preliminary tests of reliability and validity. Medical Care. 1996; 34(3): p. 220-233.

25. Walker NJ, Van Woerden HC, Kiparoglou V, Yang Y. Identifying seasonal and temporal trends in the pressures experienced by hospitals related to unscheduled care. BMC Health Services Research. 2016; 16: p. 307.

26. Molloy GJ, McGee HM, O'Neill D, Conroy RM. Loneliness and Emergency and Planned Hospitalizations in a Community Sample of Older Adults. Journal of the American Geriatrics Society. 2010; 58: p. 1538-1541.

27. Ellaway A, Wood S, MacIntyre S. Someone to talk to? The role of loneliness as a factor in the frequency of GP consultations. British Journal of General Practice. 1999; 49: p. 363-367.

28. Solé-Auró A, Alcañiz M. Educational attainment, gender and health inequalities among older adults in Catalonia (Spain). International Journal for Equity in Health. 2016; 15: p. 126.

29. Jankovic S, Stojisavljevic D, Jankovic J, Eric M, Marinkovic J. Association of socioeconomic status measured by education, and cardiovascular health: a population-based cross-sectional study. BMJ Open. 2014; 4: p. e005222.

30. Age Concern, Help the Aged. Introducing another world: Older people and digital inclusion. ; 2009.

31. Honeyman M, Dunn P, McKenna H. A digital NHS? An introduction to the digital agenda and plans for implementation. Briefing. The King's Fund; 2016.

32. Lau R, Murray E, Stevenson F. Implementing Change to Improve Access in UK General Practice: A Mixed-methods Exploratory Case Study. In SAPC Annual Scientific Meeting; 2016; Dublin. p. Talk code 3D.1.

For peer review only

Appendix

A section of the Service use diary completed by participants at baseline, 3 and 6 months. The binary Y/N question for section D was a new variable generated by assigning a "Yes" for any participant who responded "Yes" to any sub-questions in that section.

ID:

Diary Two

We would like to know about any contact you have had with services, organisations or others about your health and well-being.

Please fill in the number of contacts you have had **during the last three months** for each question listed below. If you haven't had any contact with them, please fill in a 0 (zero).

**A.** Have you needed to go to **hospital in the last 3 months?**<br>Yes ☐ Complete 1-4 below<br>No ☐ Go to Section B	
1. How many nights have you spent in hospital as an inpatient?	
2. How many days have you been admitted to a day ward in hospital without staying overnight?	
3. How many times have you had an appointment with a doctor in the hospital or have you attended a clinic appointment?	
4. How many times have you been treated at Casualty or Accident and Emergency?	
**B.** Have you seen/spoken your **GP or community nurse in the last 3 months**?<br>Yes ☐ Complete 1-7 below<br>No ☐ Go to Section C	
1. How many times have you visited your GP at their surgery or health centre?	
2. How many times has a GP visited you at home?	
3. How many times have you spoken to a GP on the phone?	
4. How many times has a nurse visited you at home?	
5. How many times have you visited a nurse at the surgery or health centre?	
6. How many times have you spoken to a nurse on the phone?	
7. How many times have you called NHS Direct?	

ID:

C. Have you contacted/been to **any other health care services in the last 3 months?**

Yes ☐ Complete 1-10 below
No ☐ Go to Section D

1. How many times have you seen a physiotherapist? (please indicate if NHS or private) ……………………………………….	
2. How many times have you seen an osteopath or chiropractor (please indicate if NHS or private)?………………………………	
3. How many times have you seen a dentist (please indicate if NHS or private)?……………………………………………………	
4. How many times have you seen someone about your sight (eg optician / optometrist?)	
5. How many times have you seen someone about your hearing (eg an audiologist/ or an ear/hearing clinic?)	
6. How many times have you been to a counselling service?	
7. How many times have you been to a smoking cessation service?	
8. How many times have you been to a chiropodist or podiatrist (foot) clinic?	
9. How many times have you contacted any emergency services (Police, Ambulance, Fire)? Please give details …………………………………………………………………….	
10. How many times have you used *another* health service contact? e.g. falls prevention service. Please give details …………………………………………………………………………	

D. Personal care
Please think about an average or typical week during the ***last 3 months***

Have you had any **paid** help (including both help arranged or paid for by social services and help arranged/paid for privately) with:

1. Washing, dressing or having a bath/shower

Yes ☐ No ☐ If yes, how many times *per week*? …………..

2. Cooking/preparing meals or shopping

Yes ☐ No ☐ If yes, how many times *per week*? ……………

3. Meals on wheels or other meal delivery service

Yes ☐ No ☐ If yes, how many times *per week*? …………..

4. Other (eg laundry, sitting service): please give details and number of times *per*

ID:

Have you had any **unpaid** help e.g. from family or friends, with:

5. Washing, dressing or having a bath/shower

Yes ☐ No ☐ If yes, how many times *per week*? ……………

6. Cooking/preparing meals or shopping

Yes ☐ No ☐ If yes, how many times *per week*? …………...

7. Other (eg laundry, sitting service): please give details and number of times *per week*?

……………………………………………………………………………………………...

For peer review only

STROBE Statement—checklist of items that should be included in reports of observational studies

"Exploring the relationship between frequent internet use and health and social care resource use in a community-based cohort of older adults"
by Caroline S. Clarke, Jeff Round, Stephen Morris, Kalpa Kharicha, John Ford, Jill Manthorpe, Steve Iliffe, Claire Goodman, Kate Walters

Manuscript ID bmjopen-2017-015839 **29 March 2017**

	Item No.	Recommendation	Page No.	Relevant text from manuscript
**Title and abstract**	1	(*a*) Indicate the study's design with a commonly used term in the title or the abstract	1	Exploring the relationship between frequent internet use and health and social care resource use in a community-based cohort of older adults
		(*b*) Provide in the abstract an informative and balanced summary of what was done and what was found	2	**Methods**<br>Participants recruited from primary care, aged over 65 and living in semi-rural or urban areas in the south of England, were followed up at 3 and 6 months after completing a comprehensive questionnaire with personalised feedback on their health and well-being. We performed logistic regression analyses to investigate relationships between frequent internet use and patterns of service use, controlling for confounding factors, and clustering by GP practice. Four categories of service use data were gathered: use of primary NHS care; secondary NHS care; other community health and social care services; and assistance with washing, shopping and meals.<br><br>**Results**<br>Our results show, in this relatively healthy population, a positive relationship (odds ratio 1.72; 95% CI 1.33 to 2.23) between frequent internet use and use of any other community-based health services (physiotherapist, osteopath/chiropractor, dentist, optician/optometrist, counselling service, smoking cessation service, chiropodist/podiatrist, emergency services, other non-specific health services), and no relationship with the other types of care. No causal relationship can be postulated due to the study's design.<br><br>**Conclusions**<br>No observed relationship between frequent internet use and primary or secondary care

For peer review only - http://bmjopen.bmj.com/site/about/guidelines.xhtml

				use was found, suggesting that older adults without internet access are not disadvantaged regarding health care utilisation. Further research should explore how older people use the internet to access healthcare, and the impact on health.
**Introduction**				
Background/rationale	2	Explain the scientific background and rationale for the investigation being reported	4	Work has been undertaken by various groups regarding complex interventions designed to alter behaviour to improve health and well-being, enabling older adults to maintain their independence and good health for longer, however there is no clear consensus on the best approaches. It has been argued that the use of technology by older people could help in maintaining health and well-being and/or assist in managing or reducing health-related resource use; similarly, other work has suggested that older adults might be disadvantaged if they do not use information and communications technology regularly.<br><br>As the majority of older people have multi-morbidities with increasingly complex health and other needs, this might influence their use of the internet in relation to their health, as well as there being differences in digital and health literacy in comparison to younger sections of the population.
Objectives	3	State specific objectives, including any prespecified hypotheses	5	The aim of this study was to examine the relationship between frequent internet use and different types of health and social care resource use, and to consider whether differences in internet use raise concerns about equity of access and use of care services by older adults.
**Methods**				
Study design	4	Present key elements of study design early in the paper	5<br><br>7	Design: Cohort study<br><br>Analysis:<br>We undertook panel logistic regression for each of the four dichotomous dependent outcome variables on service use, with the GP surgery contributing random effects. This was included as certain variables could be affected in some way by the GP practice's local policies or working practices, meaning that including these possible effects as random was the most appropriate choice. The covariates for the final multivariate regression models were chosen using the common model-selection criteria, the Akaike information criterion (AIC) and the Bayesian information criterion (BIC). Interactions

For peer review only - http://bmjopen.bmj.com/site/about/guidelines.xhtml

				between certain variables were also tested. We report odds ratios and 95% confidence intervals to investigate the relationship between frequent internet use and different types of resource use, controlling for patient characteristics described above.
Setting	5	Describe the setting, locations, and relevant dates, including periods of recruitment, exposure, follow-up, and data collection	5	Participants: A randomly sampled cohort of community-dwelling older adult participants aged 65 years and over from five general practices in two diverse regions of southern England, recruited in 2012 and followed up for 6 months as part of the WISH study.
Participants	6	(*a*) *Cohort study*—Give the eligibility criteria, and the sources and methods of selection of participants. Describe methods of follow-up<br>*Case-control study*—Give the eligibility criteria, and the sources and methods of case ascertainment and control selection. Give the rationale for the choice of cases and controls<br>*Cross-sectional study*—Give the eligibility criteria, and the sources and methods of selection of participants	5	Data collection: Potential participants were sent letters by their GPs on behalf of the study group, and 526 of the 1,550 contacted in this way responded. Of these, 454 returned the M-RAO (Multi-dimensional Risk Appraisal in Older people) questionnaire.<br><br>Further detail regarding the WISH study recruitment and data collection procedures are described elsewhere (reference 16).
		(*b*) *Cohort study*—For matched studies, give matching criteria and number of exposed and unexposed<br>*Case-control study*—For matched studies, give matching criteria and the number of controls per case	-	Not applicable
Variables	7	Clearly define all outcomes, exposures, predictors, potential confounders, and effect modifiers. Give diagnostic criteria, if applicable	5-7	Four binary resource use (dependent) variables:<br>• A. Secondary care<br>• B. Primary care<br>• C. Other health care services (either NHS or private)<br>• D. Assistance with washing/meals<br><br>Independent variable:

For peer review only - http://bmjopen.bmj.com/site/about/guidelines.xhtml

				• Internet use (frequently vs. infrequently)<br><br>Covariates:<br>• GP practice location type (urban or semi-rural)<br>• season of study entry (summer or autumn)<br>• sex<br>• age (in bands: 65-74 years, 75-84 years, 85+ years)<br>• ethnicity (White British or other)<br>• de Jong Gierveld loneliness status (scoring 0-1 or 2-6 on the de Jong Gierveld 6-item short scale corresponds to “not lonely” or “lonely”, respectively)<br>• Lubben Social Network Scale social isolation status (scoring below 12 corresponds to “socially isolated”)<br>• binary response to “Do you feel lonely much of the time?”<br>• Short Form SF-12 mental health component summary score (MCS) and physical health component summary score (PCS)<br>• occurrence of a recent sudden illness in the 3 months before baseline<br>• age at which left full-time education (before or after 17 years of age)<br>• receipt of pension (state pension only vs. other)
Data sources/ measurement	8*	For each variable of interest, give sources of data and details of methods of assessment (measurement). Describe comparability of assessment methods if there is more than one group	4-5	Participants in the WISH (Well-being Interventions for Social and Health needs) study ... were sent the Multi-dimensional Risk Appraisal in Older people (MRA-O) as a postal questionnaire. ... Participants were asked questions covering a broad range of health, lifestyle, social and environmental domains, including questions on their use of the internet.<br><br>Also see Appendix for the questions themselves, and see published WISH baseline paper (reference 16) for further details.
Bias	9	Describe any efforts to address potential sources of bias	3	Article Summary:<br>Lack of bias: the question on internet use was one of many, so participants will not have attached much weight to it and this should minimise any reporting bias.
			6	Use of binary Y/N resource use responses instead of numbers of visits:<br>This was done to minimise the amount of missing data.

For peer review only - http://bmjopen.bmj.com/site/about/guidelines.xhtml

				Also see WISH baseline paper (reference 16) for further consideration of bias in the original study.
Study size	10	Explain how the study size was arrived at	5	See WISH baseline paper (reference 16). All available data from that study were used in this analysis.
Quantitative variables	11	Explain how quantitative variables were handled in the analyses. If applicable, describe which groupings were chosen and why	6-7	Groupings:<br>internet use (frequently vs. infrequently)<br>• The internet use question offered four possible answers: often (most days); sometimes (1-3 days a week); occasionally (less than once a week); and never. For the purposes of this analysis, it was dichotomised as "often/sometimes" (frequently) vs. "occasionally/never" (infrequently) as the numbers of responses across the four groups were too small to allow meaningful analysis as a four-category variable.<br><br>age (65-74 years, 75-84 years, 85+ years)<br>• The age group variable was included as a factor variable to remove the assumption of a linear effect with age. Its joint significance was also tested using the chi-squared test.
Statistical methods	12	(*a*) Describe all statistical methods, including those used to control for confounding	7	We undertook panel logistic regression for each of the four dichotomous dependent outcome variables on service use, with the GP surgery contributing random effects. This was included as certain variables could be affected in some way by the GP practice's local policies or working practices, meaning that including these possible effects as random was the most appropriate choice. The covariates for the final multivariate regression models were chosen using the common model-selection criteria, the Akaike information criterion (AIC) and the Bayesian information criterion (BIC). Interactions between certain variables were also tested. We report odds ratios and 95% confidence intervals to investigate the relationship between frequent internet use and different types of resource use, controlling for patient characteristics described above.

For peer review only

For peer review only - http://bmjopen.bmj.com/site/about/guidelines.xhtml

				The data were set in Stata as panel data using the patient ID code as the panel variable, and the number of months' follow-up was set as the time variable (0, 3 and 6 months), although exclusion of the time variable when setting the data led to no difference in the regression results.
		(*b*) Describe any methods used to examine subgroups and interactions	7	The covariates for the final multivariate regression models were chosen using the common model-selection criteria, the Akaike information criterion (AIC) and the Bayesian information criterion (BIC). Interactions between certain variables were also tested.
		(*c*) Explain how missing data were addressed	7	Missing data<br>Demographic data were completed by all 454 participants who returned the M-RAO, except for 7 missing responses to the ethnicity question. Other questions and sub-questions were not always completed. We used complete case analysis for the four panel regression models and have not imputed any missing data. Numbers of missing data in each case are detailed in the tables below as required, with the largest proportion of missing data at baseline being 11% (50/454) in the de Jong Gierveld loneliness variable. Most variables in these analyses had much lower proportions of missing data (~2%). With such low rates of missing data, it was decided that undertaking multiple imputation to estimate new values would not be an efficient use of time. At later timepoints there were some drop-outs, leading to 89% retention at the 3-month timepoint and 77% retention at 6 months.
		(*d*) *Cohort study*—If applicable, explain how loss to follow-up was addressed<br>*Case-control study*—If applicable, explain how matching of cases and controls was addressed<br>*Cross-sectional study*—If applicable, describe analytical methods taking account of sampling strategy	7	Loss to follow-up was low. (see Missing data paragraph above)
		(*e*) Describe any sensitivity analyses		Not applicable
**Results**				
Participants	13*	(a) Report numbers of individuals at each stage of study—eg numbers potentially eligible, examined for	5	Data collection: Potential participants were sent letters by their GPs on behalf of the study group, and 526 of the 1,550 contacted in this way responded. Of these,

For peer review only - http://bmjopen.bmj.com/site/about/guidelines.xhtml

		eligibility, confirmed eligible, included in the study, completing follow-up, and analysed	7	454 returned the M-RAO.<br><br>At later timepoints there were some drop-outs, leading to 89% retention at the 3-month timepoint and 77% retention at 6 months.
		(b) Give reasons for non-participation at each stage		See WISH baseline paper (reference 16).
		(c) Consider use of a flow diagram		
Descriptive data	14*	(a) Give characteristics of study participants (eg demographic, clinical, social) and information on exposures and potential confounders	8	See Table 1
		(b) Indicate number of participants with missing data for each variable of interest	8-9	See Table 1 and Table 2.
		(c) *Cohort study*—Summarise follow-up time (eg, average and total amount)	2	Participants recruited from primary care, aged over 65 and living in semi-rural or urban areas in the south of England, were followed up at 3 and 6 months.
Outcome data	15*	*Cohort study*—Report numbers of outcome events or summary measures over time	9	See Table 2
		Case-control study—Report numbers in each exposure category, or summary measures of exposure		Not applicable
		Cross-sectional study—Report numbers of outcome events or summary measures		Not applicable
Main results	16	(*a*) Give unadjusted estimates and, if applicable, confounder-adjusted estimates and their precision (eg, 95% confidence interval). Make clear which confounders were adjusted for and why they were included	9	See Table 3 and Table 4.<br><br>Univariable unadjusted analyses<br>Shown in Table 3 are the raw unadjusted relationships between each of the covariates included as confounders in the final multi-variable models and each binary service use variable. These results show the relationship between each resource use variable and each covariate, with no controlling for any other covariate.<br><br>Multi-variable adjusted analyses<br>Models with controlling variables included were constructed using the AIC and BIC, and gave an improved fit to the data compared to the univariable models.

For peer review only - http://bmjopen.bmj.com/site/about/guidelines.xhtml

				The controlling variables included were: age, sex, site, season at start, SF-12 mental and physical component scores, having had a recent sudden illness, ethnicity, age at which left full-time education, and de Jong Gierveld loneliness status.
		(*b*) Report category boundaries when continuous variables were categorized		See Table 3 and Table 4.
		(*c*) If relevant, consider translating estimates of relative risk into absolute risk for a meaningful time period		
Other analyses	17	Report other analyses done—e.g. analyses of subgroups and interactions, and sensitivity analyses	9	Interactions between pension and internet use, between age at which left full-time education and internet use, and between binary social isolation variable derived from the Lubben Social Network Scale and binary response to "Do you feel lonely much of the time", were tested, but did not improve the model fit for any of the four regressions and so were not included.
**Discussion**				
Key results	18	Summarise key results with reference to study objectives	12	Our results show that, in this relatively healthy older adult population, there was a strong and positive relationship between frequent internet use and use of any community-based health services such as physiotherapist, osteopath/chiropractor, dentist, optician/optometrist, hearing clinic/audiologist, counsellor, smoking cessation service, chiropodist/podiatrist, and calls to the emergency services (see Appendix). Use of the internet could be implicated in a person's ability to find any of these community-based services, except perhaps the emergency services. It is not possible to infer a causal relationship between frequent internet use and community health service use based on this analysis.
			12	We did not observe disadvantages in terms of accessing primary or secondary health care in those who used the internet infrequently, although the study was not powered to detect such differences. We also did not observe a disadvantage in accessing informal assistance with washing and meals.
Limitations	19	Discuss limitations of the study, taking into account	13	Limitations of this analysis are that the sample size is relatively small, and that

For peer review only - http://bmjopen.bmj.com/site/about/guidelines.xhtml

		sources of potential bias or imprecision. Discuss both direction and magnitude of any potential bias		resource use was binary, rather than counting the number of contacts that participants had (this was due to missing responses to sub-questions regarding the numbers of specific contacts). In addition, no causality can be inferred due to the nature of the study, and we do not have comprehensive information on the reasons for participants' internet use. We cannot speculate on how much of their internet use was specifically for looking up information on health, as opposed to for example keeping in touch with family and friends, or obtaining information on transport services or tradespeople, for example. The population that took part in this study has been compared to 2011 census data, and the study population was slightly younger, more likely to be owner-occupiers, and less likely to be in an ethnic minority than the census population.
Interpretation	20	Give a cautious overall interpretation of results considering objectives, limitations, multiplicity of analyses, results from similar studies, and other relevant evidence	12	It is not possible to infer a causal relationship between frequent internet use and community health service use based on this analysis. The relationship could have arisen due to one of the following reasons: participants using the internet in order to research services that they wish to use; or participants using services being influenced by other service users or other associated factors and thereby encouraged to use the internet. However, there could equally be no relationship at all, as correlation does not imply causation: those interested in and capable of using the internet might simply also be more aware of what services are on offer.<br><br>We did not observe disadvantages in terms of accessing primary or secondary health care in those who used the internet infrequently, although the study was not powered to detect such differences. We also did not observe a disadvantage in accessing informal assistance with washing and meals. This is perhaps surprising as needing assistance with washing and meals suggests significant impairment in functioning, which might also impact on internet use. No firm conclusions can be drawn, however, as we do not know from the study what the internet use entailed; for example, if participants used the internet to find out information about their health or local health care services or otherwise.
			12-13	Statistics published by the ONS state that levels of internet use are growing ... People aged 75 years and over consistently have the lowest internet usage rates, in

For peer review only - http://bmjopen.bmj.com/site/about/guidelines.xhtml

				agreement with our observations, but these rates are increasing: from 19.9% of this age group in 2011, to 33.0% in 2015, and 38.7% in 2016. These figures suggest that the digital divide between younger and older age groups might be diminishing in terms of a simple measure of internet use.
			13	There are various initiatives under way to increase the online presence and activity of GP practices, but some concerns have been raised that this might disadvantage those who use the internet less frequently, for example some older adults, particularly women aged over 75, or other disadvantaged groups such as those with disabilities. On the other hand, it has also been postulated that use of online GP services by younger or more technologically literate patients frees up time for receptionists to respond to older adults' telephone calls. **Our results are consistent with preliminary suggestions that there might be no cause for concern regarding increasing inequity of access for older people as a whole, though there may be smaller sub-groups within this population who are adversely affected. This present study lacked sufficient power to confirm or refute this, and our patient group was a relatively healthy group, recruited via primary care**.
Generalisability	21	Discuss the generalisability (external validity) of the study results	13	Implications<br>This is one of only a few studies that has investigated internet use alongside the use of other services. Our findings were exploratory and suggest the need for further research to better understand the relationships. In the future, in order to obtain more precise information on the nature of the relationship between technology use and use of health or social care services, further detail could be asked regarding the purpose of internet use, actions taken as a result of internet access, and what type of device is used to access the internet. Online technology changes very quickly, and this study offers a timely update on its use by older people living at home. Future work should aim more to understand how older people use technology for their own healthcare both in terms of content and as a way to access information. The use of the internet by older people in long-term care facilities and in hospitals remains under-explored.

For peer review only - http://bmjopen.bmj.com/site/about/guidelines.xhtml

**Other information**				
Funding	22	Give the source of funding and the role of the funders for the present study and, if applicable, for the original study on which the present article is based	3	The WISH study was funded by the Medical Research Council (MRC) LLHW G1001822/1. The MRC had no role in the design, collection, analysis, or interpretation of data; in the writing of this manuscript; or in the decision to submit the manuscript for publication. Ethical approval for the WISH study was granted by London-East Research Ethics Committee (reference 11/LO/1814) which included permissions to conduct the analysis reported in this paper. The corresponding author for this analysis was not involved in the WISH study and received no specific grant from any funding agency in the public, commercial or not-for-profit sectors.

*Give information separately for cases and controls in case-control studies and, if applicable, for exposed and unexposed groups in cohort and cross-sectional studies.

Note: An Explanation and Elaboration article discusses each checklist item and gives methodological background and published examples of transparent reporting. The STROBE checklist is best used in conjunction with this article (freely available on the Web sites of PLoS Medicine at http://www.plosmedicine.org/, Annals of Internal Medicine at http://www.annals.org/, and Epidemiology at http://www.epidem.com/). Information on the STROBE Initiative is available at www.strobe-statement.org.

For peer review only

For peer review only - http://bmjopen.bmj.com/site/about/guidelines.xhtml

BMJ Open

Exploring the relationship between frequent internet use and health and social care resource use in a community-based cohort of older adults: an observational study in primary care

Journal:	*BMJ Open*
Manuscript ID	bmjopen-2017-015839.R2
Article Type:	Research
Date Submitted by the Author:	25-May-2017
Complete List of Authors:	Clarke, Caroline; University College London, Research Department of Primary Care and Population Health<br>Round, Jeff; University of Bristol School of Social and Community Medicine<br>Morris, Stephen; University College London, Department of Applied Health Research<br>Kharicha, Kalpa; University College London, Primary Care and Population Health<br>Ford, John; University of East Anglia, Public Health<br>Manthorpe, Jill; King's College London, Social Care Workforce Research Unit<br>Iliffe, Steve; University College London, Primary Care and Population Sciences<br>Goodman, Claire; University of Hertfordshire, Centre for Research in Primary and Community Care<br>Walters, Kate; University College London, Primary Care and Population Health
**Primary Subject Heading**:	General practice / Family practice
Secondary Subject Heading:	Health services research, Geriatric medicine
Keywords:	Older adults, Health service resource use, Internet use, Panel data, Logistic regression, PRIMARY CARE

SCHOLARONE™
Manuscripts

Exploring the relationship between frequent internet use and health and social care resource use in a community-based cohort of older adults: an observational study in primary care

Caroline S. Clarke[1], Jeff Round[2], Stephen Morris[3], Kalpa Kharicha[1], John Ford[4], Jill Manthorpe[5], Steve Iliffe[1]*, Claire Goodman[6], Kate Walters[1]

Affiliations:

[1] UCL Research Dept. of Primary Care & Population Health, UCL Medical School, Upper 3rd Floor, Royal Free Campus, Rowland Hill St, London, NW3 2PF, UK

[2] School of Social and Community Medicine, University of Bristol, Canynge Hall, 39 Whatley Road, Bristol, BS8 2PS, UK

[3] Department of Applied Health Research, University College London (UCL), 1-19 Torrington Place, London, WC1E 7HB, UK

[4] Department of Public Health and Primary Care, Norwich Medical School, University of East Anglia, Chancellors Drive, Norwich, NR4 7TJ, UK

[5] Social Care Workforce Research Unit, King's College London, Strand, London, WC2 4LF, UK

[6] Centre for Research in Primary and Community Care, University of Hertfordshire, AL10 9AB, UK

* Emeritus

Corresponding author:

Email: caroline.clarke@ucl.ac.uk

Tel.: +44 (0)20 7794 0500 ext. 36770

For peer review only

ABSTRACT

Objectives

Given many countries' ageing populations, policymakers must consider how to mitigate or reduce health problems associated with old age, within budgetary constraints. Evidence of use of digital technology in delaying the onset of illness and reducing healthcare service use is mixed, with no clear consensus as yet. Our aim was to investigate the relationship between frequent internet use and patterns of health or social care resource use in primary care attendees who took part in a study seeking to improve the health of older adults.

Methods

Participants recruited from primary care, aged over 65 and living in semi-rural or urban areas in the south of England, were followed up at 3 and 6 months after completing a comprehensive questionnaire with personalised feedback on their health and well-being. We performed logistic regression analyses to investigate relationships between frequent internet use and patterns of service use, controlling for confounding factors, and clustering by GP practice. Four categories of service use data were gathered: use of primary NHS care; secondary NHS care; other community health and social care services; and assistance with washing, shopping and meals.

Results

Our results show, in this relatively healthy population, a positive relationship (odds ratio 1.72; 95% CI 1.33 to 2.23) between frequent internet use and use of any other community-based health services (physiotherapist, osteopath/chiropractor, dentist, optician/optometrist, counselling service, smoking cessation service, chiropodist/podiatrist, emergency services, other non-specific health services), and no relationship with the other types of care. No causal relationship can be postulated due to the study's design.

Conclusions

No observed relationship between frequent internet use and primary or secondary care use was found, suggesting that older adults without internet access are not disadvantaged regarding health care utilisation. Further research should explore how older people use the internet to access healthcare, and the impact on health.

Key words: Older adults, health service resource use, internet use, panel data, logistic regression, primary care.

For peer review only - http://bmjopen.bmj.com/site/about/guidelines.xhtml

ARTICLE SUMMARY

Strengths and limitations of this study

Strengths:

- Timely study providing an update on older adults' use of the internet at home and on their use of health and other care services.
- Findings on internet use are one aspect of a survey that addressed health and social care resource use, thus being well positioned to capture the everyday experience of community-dwelling older people.

Limitations:

- We cannot speculate on how much internet use was specifically for looking up information on health or accessing health-related services, as opposed to general correspondence, or seeking information on any other non-health services, for example.
- Causality cannot be inferred.
- Small study size (n=454)

Funding: The WISH study was funded by the Medical Research Council (MRC) LLHW G1001822/1. The MRC had no role in the design, collection, analysis, or interpretation of data; in the writing of this manuscript; or in the decision to submit the manuscript for publication. Ethical approval for the WISH study was granted by London-East Research Ethics Committee (reference 11/LO/1814) which included permissions to conduct the analysis reported in this paper. The corresponding author for this analysis was not involved in the WISH study and received no specific grant from any funding agency in the public, commercial or not-for-profit sectors.

Conflict of Interest: All authors have completed the ICMJE uniform disclosure form at www.icmje.org/coi_disclosure.pdf and declare: no support from any organisation for the submitted work; no financial relationships with any organisations that might have an interest in the submitted work in the previous three years; no other relationships or activities that could appear to have influenced the submitted work.

Data sharing statement: no additional data available.

For peer review only

For peer review only - http://bmjopen.bmj.com/site/about/guidelines.xhtml

INTRODUCTION

Life expectancy is rising worldwide, and much research seeks to find ways of improving older people's health and well-being (1). Work has been undertaken by various groups regarding complex interventions designed to alter behaviour to improve health and well-being, enabling older adults to maintain their independence and good health for longer, however there is no clear consensus on the best approaches (2) (3) (4) (5). It has been argued that the use of technology by older people could help in maintaining health and well-being and/or assist in managing or reducing health-related resource use(6) (7); similarly, other work has suggested that older adults might be disadvantaged if they do not use information and communications technology regularly(8).

There is significant use of the internet by older people in the United Kingdom (UK), particularly by those in their 60s and 70s, but it is not universal and decreases with age (88.3% of 55- to 64-year-olds had used the internet in the last 3 months in 2016, 74.1% of those in the 65-74 age group, and 38.7% of those in the 75+ age group) (9). Research on how older people's use of the internet might influence the way they seek help/use healthcare and other resources is still in its infancy(10)(11)(12)(13). There seem to be differences between how younger and older people use the internet; for example, older adults who use the internet seem to use it primarily for email, whereas a large proportion of younger people use social media sites, both for information and for socialising(14). As nearly half of older people in Scotland were reported to have multi-morbidities with increasingly complex health and other needs(15), this might influence their use of the internet in relation to their health, as well as there being differences in digital and health literacy in comparison to younger sections of the population.

In the UK, some GP practices offer online services to patients, including appointment booking systems, and even online access to patients' own primary care records, although this latter example is not yet widely established. Also, health care providers are now assessed and ranked, and patients' opinions regarding services can be found online on the NHS Choices website. It is not yet clear what the uptake and impact of these various NHS online information resources are across age groups and among other sections of society, but it is conceivable that not using the internet might hinder use of these services and therefore access to health care.

Participants in the WISH (Well-being Interventions for Social and Health needs) study (16) were community-dwelling older adults recruited from English primary care settings in the London Borough of Ealing (urban) and Hertfordshire (semi-rural) and they were sent the Multi-dimensional Risk Appraisal in Older people

For peer review only - http://bmjopen.bmj.com/site/about/guidelines.xhtml

(MRA-O) as a postal questionnaire. All participants gave informed consent to participate in accordance with ethical guidelines and Good Clinical Practice. The MRA-O is an extension of the Health Risk Appraisal in Older people (HRA-O) system (17) (18) (19), including domains identified as having an impact on health and well-being in later life during the Smarter Working in Social and Health Care (SWISH) project (20) (21). Participants were asked questions covering a broad range of health, lifestyle, social and environmental domains, including questions on their use of the internet. The resource use data included information on a wide range of services, both public and privately funded, and data on use of the internet, meaning that this dataset could enable us to explore the relationship between internet use and resource use, while considering various possible confounders and adjusting for important covariates.

The aim of this study was to examine the relationship between frequent internet use and different types of health and social care resource use, and to consider whether differences in internet use raise concerns about equity of access and use of care services by older adults.

METHODS

The methods used in this analysis are compliant with the STROBE guidelines for observational cohort studies(22).

Design: Cohort study

Participants: A random sample of eligible community-dwelling older adult participants aged 65 years and over from five general practices in two diverse regions of southern England, were recruited in 2012 and followed up for 6 months as part of the WISH study (16). Random sampling was completed by the participating practices using their electronic records systems. Further information on the eligibility criteria for this study is given in previous work (16).

Data collection: Potential participants were sent letters by their GPs on behalf of the study group, and 526 of the 1,550 contacted in this way responded. Of these, 454 returned the M-RAO. The data collected included physical and mental well-being, functional ability, lifestyle and diet, personal characteristics, loneliness and social networks, use of health care and social resources, and internet and mobile phone use. Further detail regarding the WISH study recruitment and data collection procedures are described elsewhere (16).

Measurements

For peer review only - http://bmjopen.bmj.com/site/about/guidelines.xhtml

Resource use

The WISH study measured resource use across a range of services, including primary and secondary healthcare, informal and other community health care, and support from informal or family carers or social care services. These were captured in this analysis as four individual binary resource use variables, where "yes" meant that one or more of the difference types of contact listed below had occurred within the last three months:

A. Secondary care: hospital attendance (A&E, inpatient, outpatient)
B. Primary care: GP/community nurse consultation (by phone, face-to-face, a home visit, or a call to NHS Direct)
C. Other health care services (either NHS or private): physiotherapist, osteopath/chiropractor, dentist, optician/optometrist, hearing clinic/audiologist, counsellor, smoking cessation service, chiropodist/podiatrist, emergency services (police, ambulance, fire)
D. Wash/meals: any paid or unpaid help (e.g. from family member or social care services) with washing, dressing, having a bath/shower, cooking/preparing meals, shopping, or meal delivery service. The overall binary variable here returns a "yes" if any paid or unpaid help was reported.

Participants who responded 'Yes' were also asked sub-questions in each case, regarding how many contacts they had had with different services, e.g. how many nights the participant stayed in hospital, how many times they spoke with the general practice nurse on the phone. The complete list of questions can be found in the Appendix. The principal binary questions for the resource variables were used in the analysis instead of counting the numbers of contacts due to high levels of missing data in the sub-questions.

Internet use

The internet use question offered four possible answers: often (most days); sometimes (1-3 days a week); occasionally (less than once a week); and never. For the purposes of this analysis, it was dichotomised as "often/sometimes" (frequently) vs. "occasionally/never" (infrequently) as the numbers of responses across the four groups were too small to allow meaningful analysis as a four-category variable.

Covariates

We considered a wide range of patient characteristics for the analyses, including: GP practice location type (urban or semi-rural), season of study entry (summer or autumn), sex, age (in bands: 65-74 years, 75-84 years, 85+ years), ethnicity (White British or other), loneliness status (scoring 0-1 or 2-6 on the de Jong Gierveld 6-item short scale(23) corresponds to "not lonely" or "lonely", respectively), social isolation status (scoring below 12 on the Lubben Social Network Scale corresponds to "socially isolated"), binary response to "Do you feel lonely much of the time?", Short Form SF-12(24) mental health component summary score

For peer review only - http://bmjopen.bmj.com/site/about/guidelines.xhtml

For peer review only

(MCS) and physical health component summary score (PCS), occurrence of a recent sudden illness in the 3 months before baseline, age at which left full-time education (before or after 17 years of age), and receipt of pension (state pension only vs. other). GP practice location type was included because patterns of health care resource use necessarily vary according to population and practice density. Season of study entry was included as there is evidence that use of health care services is seasonal(25), and the loneliness and social isolation variables were included as there has been some research suggesting that, particularly in older adults, use of health care services can sometimes be a substitute for social contact(26)(27). The SF-12 was included as a short quality-of-life measure, and this measure is reported, as is usual, as its two components: the MCS and the PCS(24). Pension type and the age at which the participant left full-time education were included as proxy measures for socioeconomic status(28)(29). The simplicity of the ethnic group division chosen was due to low participant numbers in any non-White-British group, particularly in the semi-rural practices.

Analysis

We undertook panel logistic regression for each of the four dichotomous dependent outcome variables on service use, with the GP surgery contributing random effects. This was included as certain variables could be affected in some way by the GP practice's local policies or working practices, meaning that including these possible effects as random was the most appropriate choice. The covariates for the final multivariate regression models were chosen using the common model-selection criteria, the Akaike information criterion (AIC) and the Bayesian information criterion (BIC). Interactions between certain variables were also tested. We report odds ratios and 95% confidence intervals to investigate the relationship between frequent internet use and different types of resource use, controlling for patient characteristics described above. The age group variable was included as a factor variable to remove the assumption of a linear effect with age. Its joint significance was also tested using the chi-squared test. The data were set in Stata as panel data using the patient ID code as the panel variable, and the number of months' follow-up was set as the time variable (0, 3 and 6 months), although exclusion of the time variable when setting the data led to no difference in the regression results.

Missing data

Demographic data were completed by all 454 participants who returned the M-RAO, except for 7 missing responses to the ethnicity question. Other questions and sub-questions were not always completed. We used complete case analysis for the four panel regression models and have not imputed any missing data. Numbers of missing data in each case are detailed in the tables below, with the largest proportion of missing data at baseline being 11% (50/454) in the de Jong Gierveld loneliness variable. Most variables in these

For peer review only - http://bmjopen.bmj.com/site/about/guidelines.xhtml

analyses had much lower proportions of missing data (~2%). With such low rates of missing data, it was decided that undertaking multiple imputation to estimate new values would not be an efficient use of time. At later timepoints there were some drop-outs, leading to 89% retention at the 3-month timepoint and 77% retention at 6 months.

RESULTS

Sample baseline characteristics

Fixed patient characteristics measured at baseline for those covariates used in the final models, for the overall group and split by internet use, are given in Table 1. There was a large amount of missing data in the sub-questions regarding numbers of each specific type of contact in each of the four resource use types, with between 3.4% and 48.1% of those who responded "Yes" to the principal question failing to then state any numbers of contacts. Participants' use of the internet was asked as a four-category question: often, i.e. most days (44%); sometimes, i.e. 1-3 days a week (11%); occasionally, i.e. less than once a week (8%); and never (37%), and this was dichotomised as frequently (55%) and infrequently (45%) in the analysis.

Table 1. Baseline characteristics of participants, given for the overall group, as well as split according to whether or not they used the internet frequently.

Covariates		Overall n=454	Using internet infrequently, n=198	Using internet frequently, n=247
Site (semi-rural; other option was urban)		62.3%	63.6%	62.4%
Season at start (autumn; other option was summer)		47.6%	47.5%	48.2%
Gender (female)		52.9%	58.6%	48.6%
Age bands	65-74 years	59.9%	44.4%	73.7%
	75-84 years	33.3%	42.4%	24.3%
	6.8%	6.8%	13.1%	2.0%
White British (7 missing)		86.1%	84.4%	87.4%
Lonely (6-item de Jong Gierveld score) (50 missing)		34.9%	38.7%	31.2%
SF-12 mental score (mean, SD) (42 missing)		53.2, 8.6	52.6, 8.2	53.7, 8.9
SF-12 physical score (mean, SD) (42 missing)		43.9, 12.5	40.6, 12.7	46.5, 11.7
Recent sudden illness (12 missing)		17.0%	18.2%	15.7%
Left FT education before 17 years of age (3 missing)		60.8%	74.0%	49.6%

For peer review only

For peer review only - http://bmjopen.bmj.com/site/about/guidelines.xhtml

Retention at later timepoints

The total number of participants in the WISH study at baseline was 454, dropping to 405 (89% retention) at the 3-month timepoint and 348 (77% retention) at 6 months. The resource use variables that were recorded at each timepoint and form the panel dataset used in this analysis showed low proportions of missing values, such that only 4% or fewer participants were excluded from the complete case analyses on the basis of missing resource use data (see Table 2).

Table 2. Number of respondents at each timepoint (baseline, 3 months and 6 months), proportions of participants using each type of service at each of the three timepoints in the panel dataset, and numbers of missing values.

Resource use variable	Baseline n=454	3 months n=405	6 months n=348
(A) Secondary care – Yes	37%	40%	35%
– No	63%	60%	65%
	10 missing	2 missing	14 missing
(B) Primary care – Yes	74%	75%	71%
– No	26%	25%	29%
	11 missing	6 missing	15 missing
(C) Other health care – Yes	54%	57%	50%
– No	46%	43%	50%
	11 missing	3 missing	13 missing
(D) Wash/Meals – Yes	14%	18%	15%
– No	86%	82%	85%
	8 missing	2 missing	12 missing

Univariable unadjusted analyses

Shown in Table 3 are the raw unadjusted relationships between each of the covariates included as confounders in the final multi-variable models and each binary service use variable. These results show the relationship between each resource use variable and each covariate, with no controlling for any other covariate.

Multi-variable adjusted analyses

Models with controlling variables included were constructed using the AIC and BIC, and gave an improved fit to the data compared to the univariable models. The controlling variables included were: age, sex, site, season at start, SF-12 mental and physical component scores, having had a recent sudden illness, ethnicity,

For peer review only - http://bmjopen.bmj.com/site/about/guidelines.xhtml

age at which left full-time education, and de Jong Gierveld loneliness status. Interactions between pension and internet use, between age at which left full-time education and internet use, and between binary social isolation variable derived from the Lubben Social Network Scale and binary response to “Do you feel lonely much of the time”, were tested, but did not improve the model fit for any of the four regressions and so were not included. The multi-variable models’ results are shown in Table 4 and outlined here below.

(A) Hospital use

When controlling for age, sex, site, season at start, SF-12 mental and physical component scores, having had a recent sudden illness, ethnicity, age at which left full-time education, and loneliness, there was no observed association between hospital use and frequent internet use (OR 0.98, 95% CI 0.25 to 3.84) (see Table 4).

(B) Primary care

Use of primary care services, controlling for all the same variables, was also not associated with frequent internet use (OR 1.15, 95% CI 0.78 to 1.70) (see Table 4).

(C) Other healthcare

Frequent internet use was, however, positively associated with use of other health care services (e.g. optician, dentist, physiotherapist, etc.), when controlling for all the same variables (OR 1.72, 95% CI 1.33 to 2.23) (see Table 4).

(D) Washing/meals assistance

Of those participants who stated that they were using assistance of this nature, approximately a quarter were paying for these services. Receipt of assistance (paid or unpaid) for washing, cooking and similar tasks was not associated with frequent internet use, when controlling for all the same variables (OR 0.56, 95% CI 0.12 to 2.55) (see Table 4).

For peer review only

For peer review only - http://bmjopen.bmj.com/site/about/guidelines.xhtml

Table 3. Odds ratios (95% confidence interval) from univariable unadjusted analyses of each individual covariate and its relationship to each resource use variable in the panel dataset.

Independent variables		(A) Hospital use	(B) Primary care use	(C) Other health care services	(D) Wash/Meals assistance
Frequent internet use		0.76 (0.47 to 1.25)	0.79 (0.52 to 1.20)	1.42 (0.97 to 2.09)	0.12*** (0.05 to 0.29)
Age bands	65-74 years	reference case	reference case	reference case	reference case
	75-84 years	1.44 (0.85 to 2.44)	1.54 (0.98 to 2.42)	0.94 (0.62 to 1.42)	7.36*** (2.98 to 18.19)
	85+ years	1.55 (0.59 to 4.08)	4.76*** (1.70 to 13.36)	1.50 (0.68 to 3.34)	176.38*** (33.87 to 918.41)
Gender (male)		1.13 (0.69 to 1.84)	0.95 (0.62 to 1.43)	0.57** (0.39 to 0.83)	0.60 (0.25 to 1.41)
Site (urban)		1.35 (0.82 to 2.23)	0.95 (0.62 to 1.46)	1.40 (0.94 to 2.08)	2.76* (1.15 to 6.63)
Season at start (autumn)		0.71 (0.44 to 1.16)	0.71 (0.47 to 1.07)	0.84 (0.57 to 1.24)	2.26 (0.95 to 5.37)
SF-12 mental score		0.97 (0.94 to 1.00)	0.96** (0.93 to 0.98)	0.97** (0.94 to 0.99)	0.94* (0.89 to 0.99)
SF-12 physical score		0.94*** (0.92 to 0.96)	0.95*** (0.93 to 0.97)	0.98** (0.96 to 0.99)	0.85*** (0.82 to 0.89)
Recent sudden illness		5.07*** (2.68 to 9.59)	3.40*** (1.79 to 6.46)	3.46*** (2.00 to 5.99)	4.69** (1.54 to 14.30)
Not White British		0.84 (0.41 to 1.73)	1.31 (0.70 to 2.47)	0.77 (0.44 to 1.35)	2.79 (0.82 to 9.48)
Left full-time education aged 17 or older		1.21 (0.73 to 1.99)	1.10 (0.72 to 1.68)	2.29*** (1.55 to 3.40)	1.22 (0.51 to 2.92)
Lonely (6-item de Jong Gierveld)		1.79* (1.05 to 3.05)	1.71* (1.08 to 2.72)	1.65* (1.07 to 2.52)	5.41*** (2.12 to 13.79)

* p-value <0.05; ** p-value <0.01; *** p-value <0.001.

For peer review only

For peer review only - http://bmjopen.bmj.com/site/about/guidelines.xhtml

Table 4. Odds ratios (95% confidence interval) from multi-variable adjusted regression analyses for the four final models: one for each type of resource use in the panel dataset.

Four models →		(A) Hospital use	(B) Primary care use	(C) Other health care services	(D) Wash/Meals assistance
**Dependent variable: frequent internet use**		**0.98 (0.25 to 3.84)**	**1.15 (0.78 to 1.70)**	**1.72*** (1.33 to 2.23)**	**0.56 (0.12 to 2.55)**
Age bands	65-74 years	reference case	reference case	reference case	reference case
	75-84 years	1.50 (0.79 to 2.85)	1.35 (0.92 to 1.98)	1.08 (0.57 to 2.07)	3.44*** (1.69 to 6.97)
	85+ years	0.60 (0.15 to 2.44)	2.01 (0.54 to 7.41)	1.15 (0.45 to 2.97)	19.36*** (8.21 to 45.64)
Gender (male)		1.68* (1.01 to 2.81)	0.97 (0.68 to 1.39)	0.59*** (0.50 to 0.70)	0.69 (0.24 to 1.95)
Site (urban)		1.47* (1.07 to 2.04)	0.75 (0.37 to 1.54)	1.24 (0.84 to 1.84)	1.46 (0.60 to 3.52)
Season at start (autumn)		0.86 (0.71 to 1.05)	0.73* (0.56 to 0.95)	0.77 (0.57 to 1.04)	1.46 (0.81 to 2.63)
SF-12 mental score		1.01 (0.99 to 1.03)	0.96*** (0.95 to 0.98)	0.99 (0.94 to 1.04)	0.95 (0.91 to 1.00)
SF-12 physical score		0.94*** (0.92 to 0.96)	0.95** (0.93 to 0.98)	0.97* (0.95 to 1.00)	0.87*** (0.84 to 0.91)
Recent sudden illness		4.80*** (2.46 to 9.41)	1.89** (1.22 to 2.94)	2.27** (1.27 to 4.07)	1.42 (0.75 to 2.71)
Not White British		0.43** (0.25 to 0.75)	1.28 (0.51 to 3.18)	0.42** (0.24 to 0.72)	0.69 (0.16 to 2.95)
Left full-time education aged 17 or older		1.68*** (1.35 to 2.09)	1.23 (0.78 to 1.94)	2.72*** (2.19 to 3.39)	3.91*** (2.31 to 6.63)
Lonely (6-item de Jong Gierveld)		1.42 (0.80 to 2.51)	0.99 (0.64 to 1.54)	1.08 (0.56 to 2.06)	1.14 (0.54 to 2.39)

* p-value <0.05; ** p-value <0.01; *** p-value <0.001.

For peer review only

DISCUSSION

Our results show that, in this relatively healthy older adult population, there was a strong and positive relationship between frequent internet use and use of any community-based health services such as physiotherapist, osteopath/chiropractor, dentist, optician/optometrist, hearing clinic/audiologist, counsellor, smoking cessation service, chiropodist/podiatrist, and calls to the emergency services (see Appendix). Use of the internet could be implicated in a person's ability to find any of these community-based services, except perhaps the emergency services. It is not possible to infer a causal relationship between frequent internet use and community health service use based on this analysis. The relationship could have

For peer review only - http://bmjopen.bmj.com/site/about/guidelines.xhtml

arisen due to one of the following reasons: participants using the internet in order to research services that they wish to use; or participants using services being influenced by other service users or other associated factors and thereby encouraged to use the internet. However, there could equally be no relationship at all, as correlation does not imply causation: those interested in and capable of using the internet might simply also prefer to use the services that are on offer.

We did not observe disadvantages in terms of accessing primary or secondary health care in those who used the internet infrequently, although the study was not powered to detect such differences. We also did not observe a disadvantage in accessing informal assistance with washing and meals. This is perhaps surprising as needing assistance with washing and meals suggests significant impairment in functioning, which might also impact on internet use. No firm conclusions can be drawn, however, as we do not know from the study what the internet use entailed; for example, if participants used the internet to find out information about their health or local health and care services, or for other reasons.

Our analysis explored the situation regarding access to services that are not currently restricted to online-only access. However, some services in health care and other industries are moving towards being offered only online, and a report by Age UK(30) discusses this move towards online-only services, noting that older people and other digitally unengaged groups could potentially be left behind if they are not online. This is an important aspect to the future accessing of health care services that we have not been able to address in our analysis.

Notably, there are various initiatives under way to increase the online presence and activity of GP practices(31), and some concerns have been raised that this might disadvantage those who use the internet less frequently, for example some older adults, particularly women aged over 75, or other disadvantaged groups such as those with disabilities(9). On the other hand, it has also been postulated that use of online GP services by younger or more technologically literate patients frees up time for receptionists to respond to older adults' telephone calls (32). Our results are consistent with preliminary suggestions that there might be no cause for concern regarding increasing inequity of access for older people as a whole in the current context, though there may be smaller sub-groups within this population who are adversely affected. This present study lacked sufficient power to confirm or refute this, and our patient group was a relatively healthy group, recruited via primary care.

Several factors can contribute to the digital divide between older and younger age groups. These can include a lack of infrastructure, i.e. lack of access to broadband and/or wi-fi, as well as individual difficulties with

For peer review only - http://bmjopen.bmj.com/site/about/guidelines.xhtml

learning how to use the internet for those who are acquiring these skills in later life(33). It is also thought that, besides differences in digital and health literacy in comparison to younger sections of the population, some older people's complex co-morbidities and other needs might also influence their use of the internet in relation to their health (15). Research on how older people's use of the internet might influence the way they seek help or use healthcare and other resources is still in its infancy (10)(11)(12)(13).

Statistics published by the ONS(9) state that levels of internet use are growing, and the proportion of adults who had either never used the internet or not used it in the last 3 months had decreased by 13.3 percentage points since 2011. Women over the age of 75 have undergone the largest rise in 'recent' (i.e. having used the internet in the last 3 months) internet use since 2011, although less than a third of this group (32.6%) were recent users in 2016. People aged 75 years and over consistently have the lowest internet usage rates, in agreement with our observations, but these rates are increasing: from 19.9% of this age group in 2011, to 33.0% in 2015, and 38.7% in 2016 (9). These figures suggest that the digital divide between younger and older age groups might be diminishing in terms of a simple measure of internet use.

Similarly, a report by Age UK (34) suggested that the numbers of older adults using the internet have grown such that now more people aged over 65 have used the internet at some point in their life than have not. It is possible however that the speed at which older adults take up effective use of the internet will be slower than the speed at which some services progress to online-only access, so health care services and other industries must take care not to restrict access along these lines if they do not wish to disadvantage older adults and other digitally unengaged groups.

Limitations of this analysis are that the sample size is relatively small, and that resource use was binary, rather than counting the number of contacts that participants had made (this was due to missing responses to sub-questions regarding the numbers of specific contacts). In addition, no causality can be inferred due to the nature of the study, and we do not have comprehensive information on the reasons for participants' internet use. We cannot speculate on how much of their internet use was specifically for looking up information on health, as opposed to keeping in touch with family and friends, or obtaining information on transport services or tradespeople, for example. The population that took part in this study has been compared to 2011 census data, and the study population was slightly younger, more likely to be owner-occupiers, and less likely to be in an ethnic minority than the census population(16). The representativeness of the sample is also limited by the low questionnaire return rate of those approached via the initial letter from the GP, which meant that 29% of those initially approached chose to take part and returned the completed questionnaire at baseline.

For peer review only - http://bmjopen.bmj.com/site/about/guidelines.xhtml

Implications

This is one of only a few studies that has investigated internet use alongside the use of other services. Our findings were exploratory and suggest the need for further research to better understand the relationships. In the future, in order to obtain more precise information on the nature of the relationship between technology use and use of health or social care services, further detail could be asked regarding the purpose of internet use, actions taken as a result of internet access, and what type of device is used to access the internet. Online technology changes very quickly, and this study offers a timely update on its use by older people living at home. Future work should aim more to understand how older people use technology for their own healthcare both in terms of content and as a way to access information. The use of the internet by older people in long-term care facilities and in hospitals remains under-explored.

Contributorship Statement:

CSC, JR and SM designed the analysis, with important intellectual input from KK, JF, JM, SI, CG and KW. CSC cleaned the dataset with assistance from KK. CSC conducted the analysis and prepared the manuscript draft. All authors gave valuable input during the drafting process and approved the final manuscript.

REFERENCES

1. Oliver D, Foot C, Humphries R. Making our health and social care systems fit for an ageing population. [Online]: The King's Fund; 2014 [accessed July 2016]. Available from: http://www.kingsfund.org.uk/publications/making-our-health-and-care-systems-fit-ageing-population.

2. Beswick AD, Rees K, Dieppe P, Ayis S, Gooberman-Hill R, Horwood J, et al. Complex interventions to improve physical function and maintain independent living in elderly people: a systematic review and meta-analysis. Lancet. 2008; 371: p. 725-735.

3. Slegers K, van Boxtel MPJ, Jolles J. Computer use in older adults: Determinants and the relationship with cognitive change over a 6 year episode. Computers in Human Behaviour. 2012; 28: p. 1-10.

4. Lambert SD, Loiselle CG. Health Information–Seeking Behavior. Qualitative Health Research. 2007;(8): p. 1006-1019.

5. International Longevity Centre. A New Journey to Health – health information seeking behaviour across the generations. [Online]: ILC; 2015 [accessed July 2016]. Available from: www.ilcuk.org.uk.

6. NHS England and Tinder Foundation. Health & Digital: Reducing inequalities, improving society - An evaluation of the Widening Digital Participation programme. [Online]; 2016 [accessed July 2016]. Available from: http://www.tinderfoundation.org/our-thinking/news/widening-digital-participation-health-report-launches-today.

7. Hertzog C, Kramer AF, Wilson RS, Lindenberger U. Cognitive Development Can the Functional Capacity of Older Adults Be Preserved and Enhanced? Psychological Science in the Public Interest. 2008; 9(1): p. 1-65.

8. Xavier AJ, d'Orsi E, de Oliveira CM, Orrell M, Demakakos P, Biddulph JP, et al. English Longitudinal Study of Aging: Can Internet/E-mail Use Reduce Cognitive Decline? Journals of Gerontology. Series A, Biological sciences and

For peer review only - http://bmjopen.bmj.com/site/about/guidelines.xhtml

medical sciences. 2014; 69(9): p. 1117-1121.

9. Office for National Statistics (ONS). [Online]; 2016 [accessed September 2016]. Available from: http://www.ons.gov.uk/businessindustryandtrade/itandinternetindustry/bulletins/internetusers/2016.

10. Choi N. Relationship between health service use and health information technology use among older adults: analysis of the US National Health Interview Survey. Journal of Medical Internet Research. 2011; 13(2): p. e33.

11. Choi NG, DiNitto DM. Internet use among older adults: association with health needs, psychological capital, and social capital. Journal of Medical Internet Research. 2013; 15(5): p. e97.

12. Elliot AJ, Mooney CJ, Douthit KZ, Lynch MF. Predictors of older adults' technology use and its relationship to depressive symptoms and well-being. The Journals of Gerontology Series B: Psychological Sciences and Social Sciences. 2014; 69(5): p. 667-677.

13. Damant J, Knapp M, Freddolino P, Lombard D. Effects of digital engagement on the quality of life of older people. Health & social care in the community. 2016; DOI: 10.1111/hsc.12335.

14. Pew Research Center. Older Adults and Technology Use. [Online]; 2014 [accessed September 2016]. Available from: http://www.pewinternet.org/2014/04/03/older-adults-and-technology-use/.

15. Barnett K, Mercer SW, Norbury M, Watt G, Wyke S, Guthrie B. Epidemiology of multimorbidity and implications for health care, research, and medical education: a cross-sectional study. The Lancet. 2012; 380(9836): p. 37-43.

16. Walters K, Kharicha K, Goodman C, Handley M, Manthorpe J, Cattan M, et al. Promoting independence, health and well-being for older people: a feasibility study of computer-aided health and social risk appraisal system in primary care. BMC Family Practice. 2017; 18(47); DOI: 10.1186/s12875-017-0620-6.

17. Stuck AE, Elkuch P, Dapp U, Anders J, Iliffe S, Swift CGftPApsg. Feasibility and yield of a self-administered questionnaire for health risk appraisal in older people in three European countries. Age & Ageing. 2002; 31: p. 463-467.

18. Iliffe S, Kharicha K, Harari D, Swift C, Stuck AE. Health risk appraisal for older people in general practice using an expert system: a pilot study. Health and Social Care in the Community. 2005; 13: p. 21-29.

19. Harari D, Iliffe S, Kharicha K, Egger M, Gillman G, von Renteln-Kruse W, et al. Promotion of health in older people: an RCT of health risk appraisal in British general practice. Age & Ageing. 2008; 37(5): p. 565-571.

20. Manthorpe J, Kharicha K, Goodman C, Harari D, Swift C, Iliffe S. Smarter Working in Social and Health Care: Professional Perspectives on a New Technology for Risk Appraisal with Older People. British Journal of Social Work. 2010; 40(6): p. 1829-1846.

21. Iliffe S, Kharicha K, Harari D, Swift C, Goodman C, Manthorpe J. User involvement in the development of a health promotion technology for older people: findings from the SWISH project. Health and Social Care in the Community. 2010; 18(2): p. 147-159.

22. von Elm E, Altman DG, Egger M, Pocock SJ, Gøtzsche PC, Vandenbroucke JP, et al. The Strengthening the Reporting of Observational Studies in Epidemiology (STROBE) statement: guidelines for reporting observational studies. PLoS Medicine. 2007 Oct 16; 4(10): p. e296.

23. de Jong Gierveld J, van Tilburg T. De ingekorte schaal voor algemene, emotionele en sociale eenzaamheid [A shortened scale for overall, emotional and social loneliness]. Tijdschrift voor Gerontologie en Geriatrie. 2008; 39: p. 4-15.

For peer review only - http://bmjopen.bmj.com/site/about/guidelines.xhtml

24. Ware JJ, Kosinski M, Keller SD. A 12-Item Short-Form Health Survey: construction of scales and preliminary tests of reliability and validity. Medical Care. 1996; 34(3): p. 220-233.

25. Walker NJ, Van Woerden HC, Kiparoglou V, Yang Y. Identifying seasonal and temporal trends in the pressures experienced by hospitals related to unscheduled care. BMC Health Services Research. 2016; 16: p. 307.

26. Molloy GJ, McGee HM, O'Neill D, Conroy RM. Loneliness and Emergency and Planned Hospitalizations in a Community Sample of Older Adults. Journal of the American Geriatrics Society. 2010; 58: p. 1538-1541.

27. Ellaway A, Wood S, MacIntyre S. Someone to talk to? The role of loneliness as a factor in the frequency of GP consultations. British Journal of General Practice. 1999; 49: p. 363-367.

28. Solé-Auró A, Alcañiz M. Educational attainment, gender and health inequalities among older adults in Catalonia (Spain). International Journal for Equity in Health. 2016; 15: p. 126.

29. Jankovic S, Stojisavljevic D, Jankovic J, Eric M, Marinkovic J. Association of socioeconomic status measured by education, and cardiovascular health: a population-based cross-sectional study. BMJ Open. 2014; 4: p. e005222.

30. Age UK (Sally West). Later life in a digital world. [Accessed May 2017] Available from: http://www.ageuk.org.uk/Documents/EN-GB/For-professionals/Communities-and-inclusion/Later%20life%20in%20a%20digital%20world%20Age%20UK%202015.pdf?dtrk=true; 2015.

31. Honeyman M, Dunn P, McKenna H. A digital NHS? An introduction to the digital agenda and plans for implementation. Briefing. The King's Fund; 2016. [Accessed May 2017] Available from: https://www.kingsfund.org.uk/sites/files/kf/field/field_publication_file/A_digital_NHS_Kings_Fund_Sep_2016.pdf

32. Lau R, Murray E, Stevenson F. Implementing Change to Improve Access in UK General Practice: A Mixed-methods Exploratory Case Study. In SAPC Annual Scientific Meeting; 2016; Dublin. p. Talk code 3D.1.

33. Age Concern, Help the Aged. Introducing another world: Older people and digital inclusion. 2009. [Accessed March 2017] Available from: http://www.ageuk.org.uk/documents/en-gb/for-professionals/computers-and-technology/140_0809_introducing_another_world_older_people_and_digital_inclusion_2009_pro.pdf?dtrk=true

34. Age UK (Marcus Green and Phil Rossall). Age UK Digital Inclusion Evidence Review. 2013. [Accessed May 2017] Available from: http://www.ageuk.org.uk/Documents/EN-GB/For-professionals/Research/Age%20UK%20Digital%20Inclusion%20Evidence%20Review%202013.pdf?dtrk=true

For peer review only

For peer review only - http://bmjopen.bmj.com/site/about/guidelines.xhtml

Exploring the relationship between frequent internet use and health and social care resource use in a community-based cohort of older adults - Appendix

CS Clarke, J Round, S Morris, K Kharicha, J Ford, J Manthorpe, S Iliffe, C Goodman, K Walters

Appendix

A section of the Service use diary completed by participants at baseline, 3 and 6 months. The binary Y/N question for section D was a new variable generated by assigning a "Yes" for any participant who responded "Yes" to any sub-questions in that section.

Diary Two

We would like to know about any contact you have had with services, organisations or others about your health and well-being.

Please fill in the number of contacts you have had **during the last three months** for each question listed below. If you haven't had any contact with them, please fill in a 0 (zero).

**A.** Have you needed to go to **hospital in the last 3 months?** Yes ☐ Complete 1-4 below; No ☐ Go to Section B	
1. How many nights have you spent in hospital as an inpatient?	
2. How many days have you been admitted to a day ward in hospital without staying overnight?	
3. How many times have you had an appointment with a doctor in the hospital or have you attended a clinic appointment?	
4. How many times have you been treated at Casualty or Accident and Emergency?	
**B.** Have you seen/spoken your **GP or community nurse in the last 3 months**? Yes ☐ Complete 1-7 below; No ☐ Go to Section C	
1. How many times have you visited your GP at their surgery or health centre?	
2. How many times has a GP visited you at home?	
3. How many times have you spoken to a GP on the phone?	
4. How many times has a nurse visited you at home?	
5. How many times have you visited a nurse at the surgery or health centre?	
6. How many times have you spoken to a nurse on the phone?	
7. How many times have you called NHS Direct?	

For peer review only - http://bmjopen.bmj.com/site/about/guidelines.xhtml

Exploring the relationship between frequent internet use and health and social care resource use in a community-based cohort of older adults - Appendix

CS Clarke, J Round, S Morris, K Kharicha, J Ford, J Manthorpe, S Iliffe, C Goodman, K Walters

ID:

C. Have you contacted/been to **any other health care services in the last 3 months?**

Yes ☐ Complete 1-10 below
No ☐ Go to Section D

1. How many times have you seen a physiotherapist? (please indicate if NHS or private) ………………………………………..	
2. How many times have you seen an osteopath or chiropractor (please indicate if NHS or private)?……………………………	
3. How many times have you seen a dentist (please indicate if NHS or private)?…………………………………………………	
4. How many times have you seen someone about your sight (eg optician / optometrist?)	
5. How many times have you seen someone about your hearing (eg an audiologist/ or an ear/hearing clinic?)	
6. How many times have you been to a counselling service?	
7. How many times have you been to a smoking cessation service?	
8. How many times have you been to a chiropodist or podiatrist (foot) clinic?	
9. How many times have you contacted any emergency services (Police, Ambulance, Fire)? Please give details ………………………………………………………………………	
10. How many times have you used *another* health service contact? e.g. falls prevention service. Please give details …………………………………………………………………………	

D. Personal care
Please think about an average or typical week during the ***last 3 months***

Have you had any **paid** help (including both help arranged or paid for by social services and help arranged/paid for privately) with:

1. Washing, dressing or having a bath/shower

Yes ☐ No ☐ If yes, how many times *per week*? …………...

2. Cooking/preparing meals or shopping

Yes ☐ No ☐ If yes, how many times *per week*? ……………

3. Meals on wheels or other meal delivery service

Yes ☐ No ☐ If yes, how many times *per week*? …………...

4. Other (eg laundry, sitting service): please give details and number of times *per*

For peer review only - http://bmjopen.bmj.com/site/about/guidelines.xhtml

Exploring the relationship between frequent internet use and health and social care resource use in a community-based cohort of older adults - Appendix

CS Clarke, J Round, S Morris, K Kharicha, J Ford, J Manthorpe, S Iliffe, C Goodman, K Walters

ID:

Have you had any **unpaid** help e.g. from family or friends, with:

5. Washing, dressing or having a bath/shower

Yes ☐ No ☐ If yes, how many times *per week*? ....

6. Cooking/preparing meals or shopping

Yes ☐ No ☐ If yes, how many times *per week*? ....

7. Other (eg laundry, sitting service): please give details and number of times *per week*?

....

For peer review only

STROBE Statement—checklist of items that should be included in reports of observational studies

"Exploring the relationship between frequent internet use and health and social care resource use in a community-based cohort of older adults"
by Caroline S. Clarke, Jeff Round, Stephen Morris, Kalpa Kharicha, John Ford, Jill Manthorpe, Steve Iliffe, Claire Goodman, Kate Walters

Manuscript ID bmjopen-2017-015839 **29 March 2017**

	Item No.	Recommendation	Page No.	Relevant text from manuscript
**Title and abstract**	1	(*a*) Indicate the study's design with a commonly used term in the title or the abstract	1	Exploring the relationship between frequent internet use and health and social care resource use in a community-based cohort of older adults
		(*b*) Provide in the abstract an informative and balanced summary of what was done and what was found	2	**Methods**<br>Participants recruited from primary care, aged over 65 and living in semi-rural or urban areas in the south of England, were followed up at 3 and 6 months after completing a comprehensive questionnaire with personalised feedback on their health and well-being. We performed logistic regression analyses to investigate relationships between frequent internet use and patterns of service use, controlling for confounding factors, and clustering by GP practice. Four categories of service use data were gathered: use of primary NHS care; secondary NHS care; other community health and social care services; and assistance with washing, shopping and meals.<br><br>**Results**<br>Our results show, in this relatively healthy population, a positive relationship (odds ratio 1.72; 95% CI 1.33 to 2.23) between frequent internet use and use of any other community-based health services (physiotherapist, osteopath/chiropractor, dentist, optician/optometrist, counselling service, smoking cessation service, chiropodist/podiatrist, emergency services, other non-specific health services), and no relationship with the other types of care. No causal relationship can be postulated due to the study's design.<br><br>**Conclusions**<br>No observed relationship between frequent internet use and primary or secondary care

For peer review only - http://bmjopen.bmj.com/site/about/guidelines.xhtml

				use was found, suggesting that older adults without internet access are not disadvantaged regarding health care utilisation. Further research should explore how older people use the internet to access healthcare, and the impact on health..
**Introduction**				
Background/rationale	2	Explain the scientific background and rationale for the investigation being reported	4	Work has been undertaken by various groups regarding complex interventions designed to alter behaviour to improve health and well-being, enabling older adults to maintain their independence and good health for longer, however there is no clear consensus on the best approaches. It has been argued that the use of technology by older people could help in maintaining health and well-being and/or assist in managing or reducing health-related resource use; similarly, other work has suggested that older adults might be disadvantaged if they do not use information and communications technology regularly.<br><br>As nearly half of older people in Scotland were reported to have multi-morbidities with increasingly complex health and other needs, this might influence their use of the internet in relation to their health, as well as there being differences in digital and health literacy in comparison to younger sections of the population.
Objectives	3	State specific objectives, including any prespecified hypotheses	5	The aim of this study was to examine the relationship between frequent internet use and different types of health and social care resource use, and to consider whether differences in internet use raise concerns about equity of access and use of care services by older adults.
**Methods**				
Study design	4	Present key elements of study design early in the paper	5<br><br>7	Design: Cohort study<br><br>Analysis:<br>We undertook panel logistic regression for each of the four dichotomous dependent outcome variables on service use, with the GP surgery contributing random effects. This was included as certain variables could be affected in some way by the GP practice's local policies or working practices, meaning that including these possible effects as random was the most appropriate choice. The covariates for the final multivariate regression models were chosen using the common model-selection criteria, the Akaike information criterion (AIC) and the Bayesian information criterion (BIC). Interactions

For peer review only - http://bmjopen.bmj.com/site/about/guidelines.xhtml

				between certain variables were also tested. We report odds ratios and 95% confidence intervals to investigate the relationship between frequent internet use and different types of resource use, controlling for patient characteristics described above.
Setting	5	Describe the setting, locations, and relevant dates, including periods of recruitment, exposure, follow-up, and data collection	5	Participants: A random sample of eligible community-dwelling older adult participants aged 65 years and over from five general practices in two diverse regions of southern England, were recruited in 2012 and followed up for 6 months as part of the WISH study (16). Random sampling was completed by the participating practices using their electronic records systems.
Participants	6	(*a*) *Cohort study*—Give the eligibility criteria, and the sources and methods of selection of participants. Describe methods of follow-up<br>*Case-control study*—Give the eligibility criteria, and the sources and methods of case ascertainment and control selection. Give the rationale for the choice of cases and controls<br>*Cross-sectional study*—Give the eligibility criteria, and the sources and methods of selection of participants	5	Data collection: Potential participants were sent letters by their GPs on behalf of the study group, and 526 of the 1,550 contacted in this way responded. Of these, 454 returned the M-RAO (Multi-dimensional Risk Appraisal in Older people) questionnaire.<br><br>Further detail regarding the WISH study recruitment and data collection procedures are described elsewhere (reference 16).
		(*b*) *Cohort study*—For matched studies, give matching criteria and number of exposed and unexposed<br>*Case-control study*—For matched studies, give matching criteria and the number of controls per case	-	Not applicable
Variables	7	Clearly define all outcomes, exposures, predictors, potential confounders, and effect modifiers. Give diagnostic criteria, if applicable	5-7	Four binary resource use (dependent) variables:<br>• A. Secondary care<br>• B. Primary care<br>• C. Other health care services (either NHS or private)<br>• D. Assistance with washing/meals

For peer review only - http://bmjopen.bmj.com/site/about/guidelines.xhtml

For peer review only

				Independent variable:<br>• Internet use (frequently vs. infrequently)<br><br>Covariates:<br>• GP practice location type (urban or semi-rural)<br>• season of study entry (summer or autumn)<br>• sex<br>• age (in bands: 65-74 years, 75-84 years, 85+ years)<br>• ethnicity (White British or other)<br>• de Jong Gierveld loneliness status (scoring 0-1 or 2-6 on the de Jong Gierveld 6-item short scale(23) corresponds to "not lonely" or "lonely", respectively)<br>• Lubben Social Network Scale social isolation status (scoring below 12 corresponds to "socially isolated")<br>• binary response to "Do you feel lonely much of the time?"<br>• Short Form SF-12 mental health component summary score (MCS) and physical health component summary score (PCS)<br>• occurrence of a recent sudden illness in the 3 months before baseline<br>• age at which left full-time education (before or after 17 years of age)<br>• receipt of pension (state pension only vs. other)
Data sources/ measurement	8*	For each variable of interest, give sources of data and details of methods of assessment (measurement). Describe comparability of assessment methods if there is more than one group	4-5	Participants in the WISH (Well-being Interventions for Social and Health needs) study ... were sent the Multi-dimensional Risk Appraisal in Older people (MRA-O) as a postal questionnaire. ... Participants were asked questions covering a broad range of health, lifestyle, social and environmental domains, including questions on their use of the internet.<br><br>Also see Appendix for the questions themselves, and see published WISH baseline paper (reference 16) for further details.
Bias	9	Describe any efforts to address potential sources of bias	3	Article Summary:<br>Findings on internet use are one aspect of a survey that addressed health and social care resource use, thus being well positioned to capture the everyday experience of community-dwelling older people.

For peer review only - http://bmjopen.bmj.com/site/about/guidelines.xhtml

			6	Use of binary Y/N resource use responses instead of numbers of visits: This was done to minimise the amount of missing data.<br><br>Also see WISH baseline paper (reference 16) for further consideration of bias in the original study.
Study size	10	Explain how the study size was arrived at	5	See WISH baseline paper (reference 16). All available data from that study were used in this analysis.

Quantitative variables	11	Explain how quantitative variables were handled in the analyses. If applicable, describe which groupings were chosen and why	6	Groupings:<br>age (65-74 years, 75-84 years, 85+ years)<br>• The age group variable was included as a factor variable to remove the assumption of a linear effect with age, with the youngest group (65-74 years) as the reference case. Its joint significance was also tested using the chi-squared test.<br>internet use (frequently vs. infrequently)<br>• The internet use question offered four possible answers: often (most days); sometimes (1-3 days a week); occasionally (less than once a week); and never. For the purposes of this analysis, it was dichotomised as "often/sometimes" (frequently) vs. "occasionally/never" (infrequently) as the numbers of responses across the four groups were too small to allow meaningful analysis as a four-category variable.
Statistical methods	12	(*a*) Describe all statistical methods, including those used to control for confounding	7	We undertook panel logistic regression for each of the four dichotomous dependent outcome variables on service use, with the GP surgery contributing random effects. This was included as certain variables could be affected in some way by the GP practice's local policies or working practices, meaning that including these possible effects as random was the most appropriate choice. The covariates for the final multivariate regression models were chosen using the common model-selection criteria, the Akaike information criterion (AIC) and the Bayesian information criterion (BIC). Interactions between certain variables were also tested. We report odds ratios and 95% confidence intervals to investigate the relationship between frequent internet use and different types of resource use,

For peer review only - http://bmjopen.bmj.com/site/about/guidelines.xhtml

			controlling for patient characteristics described above.<br><br>The data were set in Stata as panel data using the patient ID code as the panel variable, and the number of months' follow-up was set as the time variable (0, 3 and 6 months), although exclusion of the time variable when setting the data led to no difference in the regression results.
	(*b*) Describe any methods used to examine subgroups and interactions	7	The covariates for the final multivariate regression models were chosen using the common model-selection criteria, the Akaike information criterion (AIC) and the Bayesian information criterion (BIC). Interactions between certain variables were also tested.
	(*c*) Explain how missing data were addressed	7-8	Missing data<br>Demographic data were completed by all 454 participants who returned the M-RAO, except for 7 missing responses to the ethnicity question. Other questions and sub-questions were not always completed. We used complete case analysis for the four panel regression models and have not imputed any missing data. Numbers of missing data in each case are detailed in the tables below, with the largest proportion of missing data at baseline being 11% (50/454) in the de Jong Gierveld loneliness variable. Most variables in these analyses had much lower proportions of missing data (~2%). With such low rates of missing data, it was decided that undertaking multiple imputation to estimate new values would not be an efficient use of time. At later timepoints there were some drop-outs, leading to 89% retention at the 3-month timepoint and 77% retention at 6 months.
	(*d*) *Cohort study*—If applicable, explain how loss to follow-up was addressed<br>*Case-control study*—If applicable, explain how matching of cases and controls was addressed<br>*Cross-sectional study*—If applicable, describe analytical methods taking account of sampling strategy	7-8	Loss to follow-up was low. (see Missing data paragraph above)
	(*e*) Describe any sensitivity analyses		Not applicable
**Results**			

For peer review only - http://bmjopen.bmj.com/site/about/guidelines.xhtml

Participants	13*	(a) Report numbers of individuals at each stage of study—eg numbers potentially eligible, examined for eligibility, confirmed eligible, included in the study, completing follow-up, and analysed	5	Data collection: Potential participants were sent letters by their GPs on behalf of the study group, and 526 of the 1,550 contacted in this way responded. Of these, 454 returned the M-RAO.
			7	At later timepoints there were some drop-outs, leading to 89% retention at the 3-month timepoint and 77% retention at 6 months.
		(b) Give reasons for non-participation at each stage		See WISH baseline paper (reference 16).
		(c) Consider use of a flow diagram		
Descriptive data	14*	(a) Give characteristics of study participants (eg demographic, clinical, social) and information on exposures and potential confounders	8	See Table 1
		(b) Indicate number of participants with missing data for each variable of interest	8-9	See Table 1 and Table 2.
		(c) *Cohort study*—Summarise follow-up time (eg, average and total amount)	2	Participants recruited from primary care, aged over 65 and living in semi-rural or urban areas in the south of England, were followed up at 3 and 6 months.
Outcome data	15*	*Cohort study*—Report numbers of outcome events or summary measures over time	9	See Table 2
		Case-control study—Report numbers in each exposure category, or summary measures of exposure		Not applicable
		Cross-sectional study—Report numbers of outcome events or summary measures		Not applicable
Main results	16	(*a*) Give unadjusted estimates and, if applicable, confounder-adjusted estimates and their precision (eg, 95% confidence interval). Make clear which confounders were adjusted for and why they were included		See Table 3 and Table 4.
			9	Univariable unadjusted analyses<br>Shown in Table 3 are the raw unadjusted relationships between each of the covariates included as confounders in the final multi-variable models and each binary service use variable. These results show the relationship between each resource use variable and each covariate, with no controlling for any other covariate.
			9-10	Multi-variable adjusted analyses

For peer review only - http://bmjopen.bmj.com/site/about/guidelines.xhtml

				Models with controlling variables included were constructed using the AIC and BIC, and gave an improved fit to the data compared to the univariable models. The controlling variables included were: age, sex, site, season at start, SF-12 mental and physical component scores, having had a recent sudden illness, ethnicity, age at which left full-time education, and de Jong Gierveld loneliness status. Interactions between pension and internet use, between age at which left full-time education and internet use, and between binary social isolation variable derived from the Lubben Social Network Scale and binary response to “Do you feel lonely much of the time”, were tested, but did not improve the model fit for any of the four regressions and so were not included. The multi-variable models’ results are shown in Table 4.
		(*b*) Report category boundaries when continuous variables were categorized		See Table 3 and Table 4.
		(*c*) If relevant, consider translating estimates of relative risk into absolute risk for a meaningful time period		
Other analyses	17	Report other analyses done—e.g. analyses of subgroups and interactions, and sensitivity analyses	10	Interactions between pension and internet use, between age at which left full-time education and internet use, and between binary social isolation variable derived from the Lubben Social Network Scale and binary response to “Do you feel lonely much of the time”, were tested, but did not improve the model fit for any of the four regressions and so were not included.
**Discussion**				
Key results	18	Summarise key results with reference to study objectives	12	Our results show that, in this relatively healthy older adult population, there was a strong and positive relationship between frequent internet use and use of any community-based health services such as physiotherapist, osteopath/chiropractor, dentist, optician/optometrist, hearing clinic/audiologist, counsellor, smoking cessation service, chiropodist/podiatrist, and calls to the emergency services (see Appendix). Use of the internet could be implicated in a person’s ability to find any of these community-based services, except perhaps the emergency services. It is not possible to infer a causal relationship between frequent internet use and

For peer review only - http://bmjopen.bmj.com/site/about/guidelines.xhtml

				community health service use based on this analysis.
			13	We did not observe disadvantages in terms of accessing primary or secondary health care in those who used the internet infrequently, although the study was not powered to detect such differences. We also did not observe a disadvantage in accessing informal assistance with washing and meals.
Limitations	19	Discuss limitations of the study, taking into account sources of potential bias or imprecision. Discuss both direction and magnitude of any potential bias	14	Limitations of this analysis are that the sample size is relatively small, and that resource use was binary, rather than counting the number of contacts that participants had made (this was due to missing responses to sub-questions regarding the numbers of specific contacts). In addition, no causality can be inferred due to the nature of the study, and we do not have comprehensive information on the reasons for participants' internet use. We cannot speculate on how much of their internet use was specifically for looking up information on health, as opposed to keeping in touch with family and friends, or obtaining information on transport services or tradespeople, for example. The population that took part in this study has been compared to 2011 census data, and the study population was slightly younger, more likely to be owner-occupiers, and less likely to be in an ethnic minority than the census population. The representativeness of the sample is also limited by the low questionnaire return rate of those approached via the initial letter from the GP, which meant that 29% of those initially approached chose to take part and returned the completed questionnaire at baseline.
Interpretation	20	Give a cautious overall interpretation of results considering objectives, limitations, multiplicity of analyses, results from similar studies, and other relevant evidence	12-13	It is not possible to infer a causal relationship between frequent internet use and community health service use based on this analysis. The relationship could have arisen due to one of the following reasons: participants using the internet in order to research services that they wish to use; or participants using services being influenced by other service users or other associated factors and thereby encouraged to use the internet. However, there could equally be no relationship at all, as correlation does not imply causation: those interested in and capable of using the internet might simply also prefer to use the services that are on offer.
			13	We did not observe disadvantages in terms of accessing primary or secondary

For peer review only - http://bmjopen.bmj.com/site/about/guidelines.xhtml

				health care in those who used the internet infrequently, although the study was not powered to detect such differences. We also did not observe a disadvantage in accessing informal assistance with washing and meals. This is perhaps surprising as needing assistance with washing and meals suggests significant impairment in functioning, which might also impact on internet use. No firm conclusions can be drawn, however, as we do not know from the study what the internet use entailed; for example, if participants used the internet to find out information about their health or local health and care services, or for other reasons.
			14	Statistics published by the ONS state that levels of internet use are growing ... People aged 75 years and over consistently have the lowest internet usage rates, in agreement with our observations, but these rates are increasing: from 19.9% of this age group in 2011, to 33.0% in 2015, and 38.7% in 2016. These figures suggest that the digital divide between younger and older age groups might be diminishing in terms of a simple measure of internet use.
			13	There are various initiatives under way to increase the online presence and activity of GP practices, and some concerns have been raised that this might disadvantage those who use the internet less frequently, for example some older adults, particularly women aged over 75, or other disadvantaged groups such as those with disabilities. On the other hand, it has also been postulated that use of online GP services by younger or more technologically literate patients frees up time for receptionists to respond to older adults' telephone calls. Our results are consistent with preliminary suggestions that there might be no cause for concern regarding increasing inequity of access for older people as a whole in the current context, though there may be smaller sub-groups within this population who are adversely affected. This present study lacked sufficient power to confirm or refute this, and our patient group was a relatively healthy group, recruited via primary care.
Generalisability	21	Discuss the generalisability (external validity) of the study results	15	Implications<br>This is one of only a few studies that has investigated internet use alongside the

				use of other services. Our findings were exploratory and suggest the need for further research to better understand the relationships. In the future, in order to obtain more precise information on the nature of the relationship between technology use and use of health or social care services, further detail could be asked regarding the purpose of internet use, actions taken as a result of internet access, and what type of device is used to access the internet. Online technology changes very quickly, and this study offers a timely update on its use by older people living at home. Future work should aim more to understand how older people use technology for their own healthcare both in terms of content and as a way to access information. The use of the internet by older people in long-term care facilities and in hospitals remains under-explored.
**Other information**				
Funding	22	Give the source of funding and the role of the funders for the present study and, if applicable, for the original study on which the present article is based	3	The WISH study was funded by the Medical Research Council (MRC) LLHW G1001822/1. The MRC had no role in the design, collection, analysis, or interpretation of data; in the writing of this manuscript; or in the decision to submit the manuscript for publication. Ethical approval for the WISH study was granted by London-East Research Ethics Committee (reference 11/LO/1814) which included permissions to conduct the analysis reported in this paper. The corresponding author for this analysis was not involved in the WISH study and received no specific grant from any funding agency in the public, commercial or not-for-profit sectors.

*Give information separately for cases and controls in case-control studies and, if applicable, for exposed and unexposed groups in cohort and cross-sectional studies.

Note: An Explanation and Elaboration article discusses each checklist item and gives methodological background and published examples of transparent reporting. The STROBE checklist is best used in conjunction with this article (freely available on the Web sites of PLoS Medicine at http://www.plosmedicine.org/, Annals of Internal Medicine at http://www.annals.org/, and Epidemiology at http://www.epidem.com/). Information on the STROBE Initiative is available at www.strobe-statement.org.

For peer review only - http://bmjopen.bmj.com/site/about/guidelines.xhtml

For peer review only
